# Elevated remnant cholesterol and atherosclerotic cardiovascular disease in diabetes: a population-based prospective cohort study

**DOI:** 10.1007/s00125-023-06016-0

**Published:** 2023-09-30

**Authors:** Benjamin N. Wadström, Kasper M. Pedersen, Anders B. Wulff, Børge G. Nordestgaard

**Affiliations:** 1 Department of Clinical Biochemistry, Herlev and Gentofte Hospital, Copenhagen University Hospital, Herlev, Denmark; 2 The Copenhagen General Population Study, Herlev and Gentofte Hospital, Copenhagen University Hospital, Herlev, Denmark; 3 Department of Clinical Medicine, Faculty of Health and Medical Sciences, University of Copenhagen, Copenhagen, Denmark

**Keywords:** Impaired glucose tolerance, Insulin resistance, Lower-extremity arterial disease, Triglyceride-rich lipoprotein, Very-low-density lipoprotein

## Abstract

**Aims/hypothesis:**

Elevated remnant cholesterol is observationally and causally associated with increased risk of atherosclerotic cardiovascular disease (ASCVD) in the general population. This association is not well studied in individuals with diabetes, who are often included in clinical trials of remnant cholesterol-lowering therapy. We tested the hypothesis that elevated remnant cholesterol is associated with increased risk of ASCVD in individuals with diabetes. We also explored the fraction of excess risk conferred by diabetes which can be explained by elevated remnant cholesterol.

**Methods:**

We included 4569 white Danish individuals with diabetes (58% statin users) nested within the Copenhagen General Population Study (2003–2015). The ASCVDs peripheral artery disease, myocardial infarction and ischaemic stroke were extracted from national Danish health registries without losses to follow-up. Remnant cholesterol was calculated from a standard lipid profile.

**Results:**

During up to 15 years of follow-up, 236 individuals were diagnosed with peripheral artery disease, 234 with myocardial infarction, 226 with ischaemic stroke and 498 with any ASCVD. Multivariable adjusted HR (95% CI) per doubling of remnant cholesterol was 1.6 (1.1, 2.3; *p*=0.01) for peripheral artery disease, 1.8 (1.2, 2.5; *p*=0.002) for myocardial infarction, 1.5 (1.0, 2.1; *p*=0.04) for ischaemic stroke, and 1.6 (1.2, 2.0; *p*=0.0003) for any ASCVD. Excess risk conferred by diabetes was 2.5-fold for peripheral artery disease, 1.6-fold for myocardial infarction, 1.4-fold for ischaemic stroke and 1.6-fold for any ASCVD. Excess risk explained by elevated remnant cholesterol and low-grade inflammation was 14% and 8% for peripheral artery disease, 26% and 16% for myocardial infarction, 34% and 34% for ischaemic stroke, and 24% and 18% for any ASCVD, respectively. LDL-cholesterol did not explain excess risk, as it was not higher in individuals with diabetes. We also explored the fraction of excess risk conferred by diabetes which can be explained by elevated remnant cholesterol.

**Conclusions/interpretation:**

Elevated remnant cholesterol was associated with increased risk of ASCVD in individuals with diabetes. Remnant cholesterol and low-grade inflammation explained substantial excess risk of ASCVD conferred by diabetes. Whether remnant cholesterol should be used as a treatment target remains to be determined in randomised controlled trials.

**Graphical Abstract:**

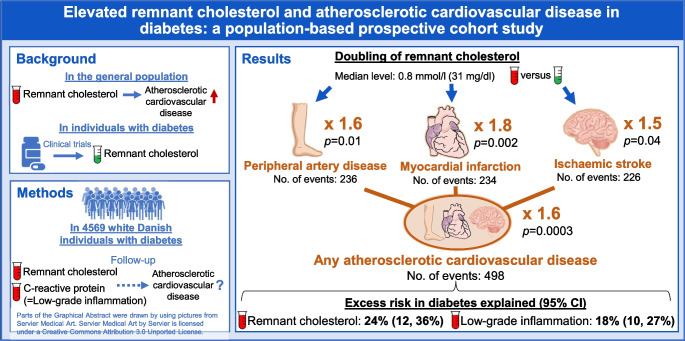

**Supplementary Information:**

The online version contains peer-reviewed but unedited supplementary material available at 10.1007/s00125-023-06016-0.


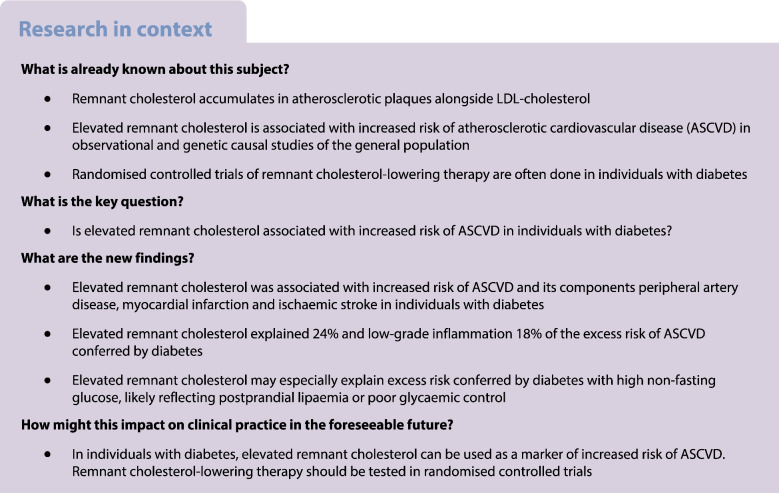


## Introduction

Diabetes will affect more than 500 million people worldwide during the 2020s [1], challenging current strategies for prevention of atherosclerotic cardiovascular disease (ASCVD). In this context, elevated remnant cholesterol, including cholesterol carried in VLDL and chylomicron remnants, is emerging as a causal risk factor for ASCVD [2, 3], in addition to elevated LDL-cholesterol. Remnant cholesterol is highly correlated with plasma triglycerides as they are both carried in triglyceride-rich lipoproteins, but since it is cholesterol that accumulates in the arterial intima, remnant cholesterol is what is relevant for risk of ASCVD [2]. Studies of the general population have shown that risk of myocardial infarction, ischaemic stroke and peripheral artery disease is increased in individuals with elevated remnant cholesterol [4–6].

Individuals with diabetes compared to individuals without diabetes have excess risk of ASCVD, which includes peripheral artery disease, myocardial infarction and ischaemic stroke, even in the absence of established risk factors such as hypertension and elevated LDL-cholesterol [7, 8]. Remnant cholesterol is often markedly elevated in individuals with diabetes due to metabolic changes caused by hyperglycaemia and insulin resistance [9]. It is therefore highly likely that elevated remnant cholesterol contributes to excess risk of peripheral artery disease, myocardial infarction and ischaemic stroke in individuals with diabetes [7, 8].

We tested the hypothesis that elevated remnant cholesterol is associated with increased risk of peripheral artery disease, myocardial infarction, ischaemic stroke and any ASCVD in individuals with diabetes.

## Methods

### Study population

We included 107,243 white Danish individuals aged 20–100 years with information on lipid levels and statin use from the Copenhagen General Population Study, which consists of individuals randomly recruited from the Copenhagen general population in 2003–2015. Among these individuals, 4569 had diabetes as defined by the criteria below. At baseline, participants completed a questionnaire, underwent a physical examination and had blood drawn for biochemical analyses. The study was approved by a local institutional review board and a Danish ethical committee (H-KF-01-144/01). All participants signed written informed consent, and the study was conducted in accordance with the Declaration of Helsinki.

### Diabetes

Individuals were defined as having diabetes if there was a diagnosis of diabetes mellitus type 1 or 2 (ICD-8 codes 249–250 and ICD-10 codes E10–E14 [http://apps.who.int/classifications/icd10/browse/2016/en]) in the national Danish Patient Registry before the baseline examination, or on the basis of self-reported diabetes, use of glucose-lowering medications or a non-fasting plasma glucose >11 mmol/l (198 mg/dl) at the baseline examination.

The national Danish Patient Registry records all medical diagnoses entered from public and private hospitals until 1994 in ICD-8, and additionally includes outpatient and emergency departments from 1995 in ICD-10. Type 2 diabetes was classified as diabetes after excluding individuals with any diagnosis indicative of type 1 diabetes (ICD-8 code 249 and ICD-10 code E10). Type 1 diabetes was classified as a first diagnosis indicative of diabetes mellitus type 1 in the national Danish Patient Registry before age 45 years [10], as only a small minority of newly diagnosed diabetes at higher ages represent diabetes mellitus type 1. Type ambiguity was a first diagnosis indicative of diabetes mellitus type 1 at or after age 45 years.

### Endpoints

Individuals living in Denmark have since 1968 been assigned a Central Person Registration number at birth or immigration through the national Danish Civil Registration System, allowing linkage to nationwide Danish health registries, ensuring no losses to follow-up.

Peripheral artery disease diagnoses were from the national Danish Patient Registry (ICD-8 codes 44020, 44029, 44030, 44389, 44399, 44500, 44509, 44590; ICD-10 codes I70.2, I70.2A I73.9, I73.9A-C) [6]; myocardial infarction (ICD-8 code 410; ICD-10 codes I21–I22) and ischaemic stroke (ICD-8 codes 433–434; ICD-10 code I63) were from the national Danish Patient Registry and the national Danish Causes of Death Registry. Any ASCVD was peripheral artery disease, myocardial infarction and/or ischaemic stroke. The national Danish Patient Registry has a 69% positive predictive value for peripheral artery disease [1] and 99% for myocardial infarction [2], while diagnoses of ischaemic stroke were individually validated by two independent medical doctors reviewing medical records [3].

All individuals were followed until emigration (*n*=3), death (*n*=1182) or first event, up until 13 December 2018. Data on vital status were extracted from the Danish Civil Registration System, which is 100% complete. Individuals with an event before baseline were excluded from the corresponding analyses but could still be included in the analyses of the other endpoints.

### Laboratory methods

Non-fasting plasma triglycerides, total cholesterol, HDL-cholesterol, high-sensitivity C-reactive protein and glucose were measured using standard hospital assays. LDL-cholesterol was calculated using the Friedewald equation [11], unless triglycerides were >352 mg/dl (4 mmol/l), where it was measured directly using a standard hospital assay. Remnant cholesterol was total cholesterol minus LDL-cholesterol minus HDL-cholesterol. In 1906 of the individuals with diabetes, remnant cholesterol, LDL-cholesterol and LDL triglycerides were measured directly using NMR spectroscopy [12]. Remnant cholesterol was the cholesterol in all VLDL subfractions using this method.

### Other covariates

Information on ethnicity, gender, smoking and medication use was self-reported at baseline and were not tracked during follow-up. Use of lipid-lowering medications was >99% statins. Smoking status was recorded as never, former or current smoking, and cumulative smoking as number of pack-years smoked. Date of birth and sex were obtained from the Danish Civil Registration System. Weight and height for calculation of BMI and BP were measured at the baseline examination. Glucose-lowering medication use was insulin, other glucose-lowering medication, or both insulin and other glucose-lowering medication.

### Statistical analyses

More detailed description of the statistical analysis is given in the electronic supplementary material (ESM) Methods.

Information on covariates was 99.5% complete and missing values were imputed using a single imputation based on predictive mean matching. If individuals with incomplete data were excluded, results were similar to those presented.

Cox regression with age as timescale and left truncation at study entry (ESM Methods: Age with left truncation as timescale) was used to yield HRs of peripheral artery disease, myocardial infarction, ischaemic stroke and any ASCVD per doubling of remnant cholesterol and LDL-cholesterol, and for diabetes vs no diabetes. The proportional hazards assumption was checked using Schoenfeld residuals and log(time) plots and there were no violations. Unadjusted Poisson regression with log(follow-up time) as offset was used to estimate number of events per 1000 person-years for the median and double the median of remnant cholesterol and LDL-cholesterol. Multivariable adjustment in diabetes vs no diabetes was for age (as underlying timescale), sex, smoking status, cumulative smoking and birth year. Remnant cholesterol and LDL-cholesterol analyses were additionally adjusted for systolic BP, diastolic BP, non-fasting plasma glucose and LDL-cholesterol (in remnant cholesterol analyses) or remnant cholesterol (in LDL-cholesterol analyses). Variables that are located within the biological pathway from remnant cholesterol to ASCVD, namely BMI (remnant cholesterol and LDL-cholesterol mediate increased risk of ischaemic heart disease in obesity [13]), statin use and ASCVD before baseline, were purposely not adjusted for. Statin treatment affects remnant cholesterol and LDL-cholesterol levels, and current smoking is an important risk factor for peripheral artery disease, myocardial infarction and ischaemic stroke. Therefore, all analyses were stratified by statin use and current smoking. Due to the high number of statistical tests with varying degrees of interdependency, no threshold for significance was set. However, *p* values corresponding to *p*<0.05 Bonferroni-corrected for multiple testing were calculated for each analysis to help the reader interpret the results.

Analyses were stratified by sex and sensitivity analyses were used to test for impact of: confounding; competing risk of death; choice of timescale; measurement error; equation used to calculate remnant cholesterol and LDL-cholesterol; directly measured remnant cholesterol, LDL-cholesterol and LDL triglycerides; exclusion of individuals with type 1 diabetes; exclusion of individuals with ASCVD before baseline; and stratified by non-fasting plasma glucose (ESM Methods: Sensitivity analysis).

Explained excess risk in the pathway from diabetes to risk of peripheral artery disease, myocardial infarction, ischaemic stroke and any ASCVD was estimated from regression-based mediation analysis using the CMAverse package (https://github.com/BS1125/CMAverse/) version 0.1.0 for R [14] (ESM Methods: Analysis of explained excess risk). Cox regression with the delta method was used, adjusted for age, sex, smoking status, cumulative smoking and birth year. Remnant cholesterol, LDL-cholesterol and low-grade inflammation were each tested as explanatory factors with interaction with diabetes status. Estimates and confidence intervals below 0% were truncated to 0%. Sensitivity analysis was carried out for explained excess risk from diabetes with non-fasting plasma glucose levels either < median or ≥ median.

Correction for measurement error was carried out for remnant cholesterol and LDL-cholesterol in all regression analyses based on two repeat measurements 10 years apart in the Copenhagen City Heart Study (ESM Methods: Measurement error correction).

All statistical analyses were carried out using ‘R: A language and environment for statistical computing’ (R Foundation for Statistical Computing, Vienna, Austria), version 4.2.2 for Windows 64-bit.

## Results

### Baseline characteristics

We followed 4569 individuals with diabetes from the Copenhagen General Population Study for up to 15 years (median 8 years), during which time 236 individuals were diagnosed with peripheral artery disease, 234 with myocardial infarction, 226 with ischaemic stroke and 498 with any ASCVD (some individuals have multiple events). Baseline characteristics by remnant cholesterol levels are shown in Table 1, by statin use in ESM Table 1, by smoking status in ESM Table 2 and by diabetes type in ESM Table 3.

**Table 1:** Baseline characteristics of individuals with diabetes in the Copenhagen General Population Study grouped by levels of remnant cholesterol

	Remnant cholesterol		
Variable	<1.0 mmol/l (<39 mg/dl)	≥1.0 mmol/l (≥39 mg/dl)	All
Number of individuals	2975	1594	4569
Men	1675 (56)	957 (60)	2632 (58)
Age, years	67 (60–74)	66 (59–72)	67 (60–73)
Remnant cholesterol, mmol/l	0.6 (0.4–0.8)	1.3 (1.1–1.6)	0.8 (0.5–1.2)
Remnant cholesterol, mg/dl	24 (17–31)	52 (44–61)	31 (21–45)
LDL-cholesterol, mmol/l	2.3 (1.8–2.9)	2.5 (1.8–3.3)	2.3 (1.8–3.1)
LDL-cholesterol, mg/dl	89 (68–113)	97 (72–128)	90 (70–120)
Systolic BP, mmHg	146 (132–159)	146 (135–160)	146 (134–160)
Current smokers	495 (17)	329 (21)	824 (18)
Cumulative smoking, pack-years	9 (0–30)	18 (0–38)	11 (0–34)
Non-fasting plasma glucose, mmol/l	6.2 (5.2–8.1)	7.2 (5.8–9.7)	6.5 (5.4–8.6)
Non-fasting plasma glucose, mg/dl	112 (94–146)	130 (104–175)	117 (97–155)
C-reactive protein, mg/l	1.6 (1.0–3.0)	2.1 (1.3–4.0)	1.7 (1.1–3.3)
Within biological pathway			
Statin use	1744 (59)	899 (56)	2643 (58)
BMI, kg/m^2^	28 (25–31)	30 (27–34)	29 (26–32)
ASCVD	376 (13)	229 (14)	605 (13)
Hypertension	2489 (84)	1416 (89)	3905 (85)
Triglycerides, mmol/l	1.4 (1.0–1.8)	3.1 (2.6–3.9)	1.8 (1.2–2.7)
Triglycerides, mg/dl	120 (89–156)	277 (229–348)	159 (106–238)
Only insulin use	494 (17)	92 (6)	586 (13)
Only other glucose-lowering medication use	1311 (44)	840 (53)	2151 (47)
Both insulin and other glucose-lowering medication use	227 (8)	154 (10)	381 (8)
Diabetes characteristics			
Diabetes type 1 diagnosis	604 (20)	174 (11)	778 (17)
Diabetes type 2 diagnosis	1432 (48)	747 (47)	2179 (48)
Self-reported diabetes	2617 (88)	1365 (86)	3982 (87)
Non-fasting plasma glucose >11 mmol/l (198 mg/dl)	326 (11)	289 (18)	615 (13)

Values are median (IQR) for continuous variables and *n* (%) for categorical variables

Statin use indicates use of lipid-lowering medications, which was mostly statins. ASCVD indicates atherosclerotic cardiovascular disease, comprising peripheral artery disease, myocardial infarction and ischaemic stroke before baseline

### Remnant and LDL-cholesterol in diabetes

**Fig. 1:**
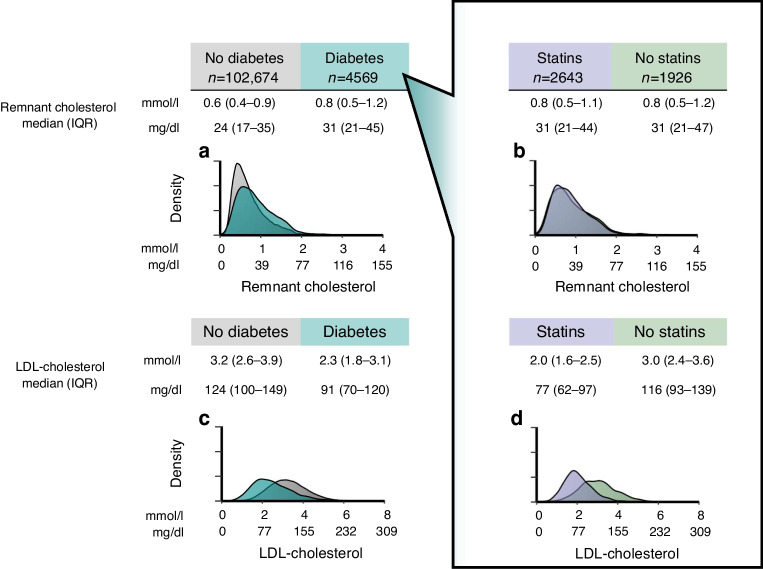
Remnant cholesterol (**a**, **b**) and LDL-cholesterol (**c**, **d**) levels of individuals in the Copenhagen General Population Study grouped by diabetes status (**a**, **c**), and by statin use in individuals with diabetes within the study (**b**, **d**). Individuals with diabetes had higher levels of remnant cholesterol, but lower levels of LDL-cholesterol, compared to individuals without diabetes

Compared to 102,674 individuals without diabetes, individuals with diabetes had higher remnant cholesterol levels but lower LDL-cholesterol levels (Fig. 1). In individuals with diabetes, remnant cholesterol levels were similar regardless of statin use, while LDL-cholesterol levels were lower in individuals using statins compared with those not using statins (Fig. 1). Remnant cholesterol and LDL-cholesterol were weakly correlated (*R*^2^=2.4%; ESM Fig. 1).

### ASCVD per doubling of remnant cholesterol and LDL-cholesterol

**Fig. 2:**
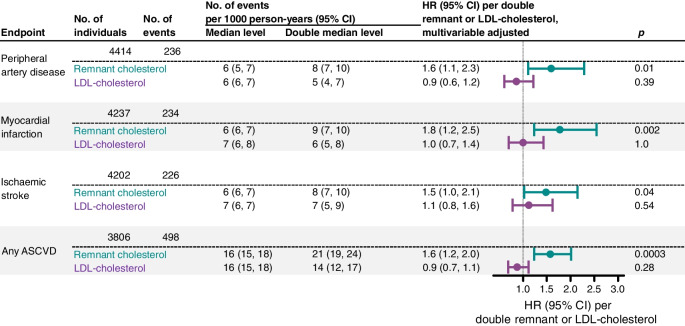
Risk of ASCVD per doubling of remnant cholesterol and LDL-cholesterol in individuals with diabetes from the Copenhagen General Population Study. Higher remnant cholesterol, but not LDL-cholesterol, was associated with increased risk of ASCVD and its components peripheral artery disease, myocardial infarction and ischaemic stroke in individuals with diabetes. Results are from Cox regression adjusted for age, sex, systolic BP, diastolic BP, smoking status, cumulative smoking, birth year, non-fasting plasma glucose and LDL-cholesterol (in remnant cholesterol analyses) or remnant cholesterol (in LDL-cholesterol analyses). The Bonferroni-corrected threshold equivalent to *p*=0.05 is 0.05/8=0.00625 due to eight statistical tests. Number of events per 1000 person-years is from unadjusted Poisson regression. No., number

In individuals with diabetes, multivariable adjusted HRs (95% CI) for risk of peripheral artery disease were 1.6 (1.1, 2.3) per doubling of remnant cholesterol and 0.9 (0.6, 1.2) per doubling of LDL-cholesterol (Fig. 2). Corresponding HRs were 1.8 (1.2, 2.5) and 1.0 (0.7, 1.4) for myocardial infarction, 1.5 (1.0, 2.1) and 1.1 (0.8, 1.6) for ischaemic stroke, and 1.6 (1.2, 2.0) and 0.9 (0.7, 1.1) for any ASCVD, respectively. These results were largely similar in statin users and non-users alike and in non-smokers and smokers alike (ESM Fig. 2 and ESM Fig. 3).

### Sensitivity analyses

Results were similar after additional adjustment for BMI and ASCVD before baseline, in competing risk regression with death as competing event, when using time in study as timescale, when additionally adjusting for high-sensitivity C-reactive protein, glucose-lowering medication use, BMI and HDL-cholesterol, when using different equations to calculate LDL and remnant cholesterol, and after excluding individuals with type 1 diabetes (ESM Fig. 4–8). As expected, estimates were attenuated without correction for regression dilution bias (ESM Fig. 4). When additionally adjusting for apolipoprotein B, estimates were similar for risk of ischaemic stroke and any ASCVD, attenuated for risk of peripheral artery disease, and strengthened for risk of myocardial infarction (ESM Fig. 6). When adjusting for plasma triglycerides, the confidence intervals were wider, as expected because of high correlation between remnant cholesterol and triglycerides (ESM Fig. 6). After excluding individuals with ASCVD before baseline, the association between elevated remnant cholesterol and risk of peripheral artery disease was slightly attenuated (ESM Fig. 8). When adjusting for LDL triglycerides measured in about half of the included individuals, estimates except for risk of myocardial infarction attenuated to the null (ESM Fig. 9). Estimates were attenuated in individuals with non-fasting plasma glucose below the median (ESM Fig. 10). Directly measured remnant cholesterol was less strongly associated with risk of peripheral artery disease and myocardial infarction but had a similar association with ischaemic stroke (ESM Fig. 11). Results for directly measured LDL-cholesterol were similar to calculated LDL-cholesterol (ESM Fig. 11). Results for directly measured remnant cholesterol were attenuated to the null after adjustment for LDL triglycerides; likewise, LDL triglycerides were not associated with risk of ASCVD after adjustment for directly measured remnant cholesterol (ESM Fig. 12). Results were similar in men and women (ESM Fig. 13).

### Excess risk of ASCVD in diabetes

**Fig. 3:**
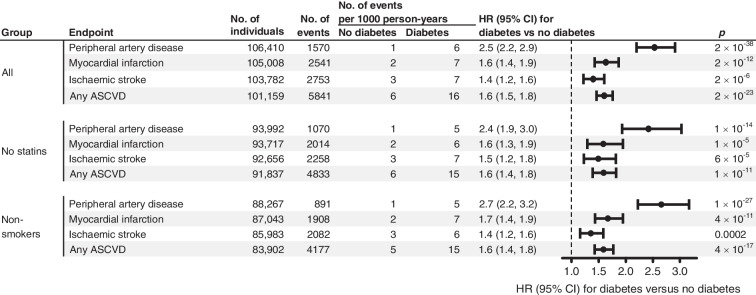
Excess risk of ASCVD in individuals with diabetes from the Copenhagen General Population Study. Individuals with diabetes had higher risk of ASCVD and its components peripheral artery disease, myocardial infarction and ischaemic stroke, compared to individuals without diabetes. Risk of peripheral artery disease was particularly increased. Results were similar when statin users and smokers were excluded. Results are from Cox regressions adjusted for age, sex, smoking status, cumulative smoking and birth year. The Bonferroni-corrected threshold equivalent to *p*=0.05 is 0.05/12= 0.0042 due to 12 statistical tests. No., number

In all individuals, the HR (95% CI) for risk of peripheral artery disease was 2.5 (2.2, 2.9) for diabetes vs no diabetes (Fig. 3). Corresponding HRs were 1.6 (1.4, 1.9) for myocardial infarction, 1.4 (1.2, 1.6) for ischaemic stroke, and 1.6 (1.5, 1.8) for any ASCVD. Results were similar in statin non-users, non-smokers (Fig. 3) and in smokers (ESM Fig. 14). In statin users, all HRs were attenuated (ESM Fig. 14).

### Explained excess risk of ASCVD

In individuals with diabetes, levels of remnant cholesterol and high-sensitivity C-reactive protein were higher compared to individuals without diabetes (ESM Fig. 15). Levels of LDL-cholesterol were lower in individuals with diabetes, also when comparing only within statin non-users or non-smokers and within statin users or smokers (ESM Fig. 15). LDL-cholesterol levels remained lower also after additional adjustment for age, sex, smoking status and cumulative smoking (data not shown). Because of this, elevated LDL-cholesterol was not tested for in the analysis of explained excess risk.

**Fig. 4:**
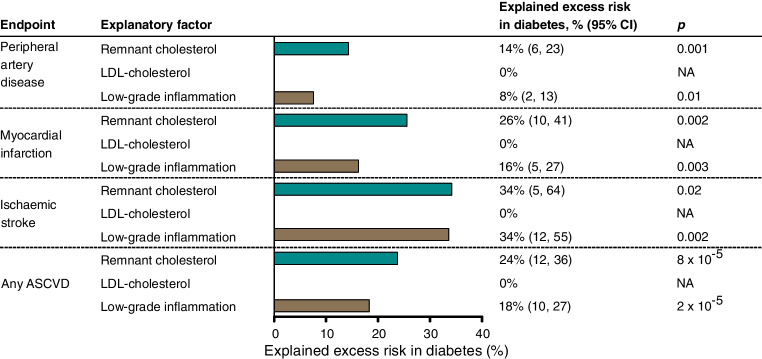
Explained excess risk of ASCVD in diabetes by remnant cholesterol, LDL-cholesterol and low-grade inflammation in the Copenhagen General Population Study. Excess risk of ASCVD and its components peripheral artery disease, myocardial infarction and ischaemic stroke in individuals with diabetes could be partly explained by elevated remnant cholesterol and low-grade inflammation measured as elevated high-sensitivity C-reactive protein, but not by elevated LDL-cholesterol. LDL-cholesterol was lower in individuals with diabetes than in individuals without diabetes and could therefore not explain excess risk. Results are from Cox regressions adjusted for age, sex, smoking status, cumulative smoking and birth year. The Bonferroni-corrected threshold equivalent to *p*=0.05 is 0.05/12= 0.0042 due to 12 statistical tests. NA, not applicable

In diabetes, elevated remnant cholesterol and low-grade inflammation explained 14% (95% CI 6%, 23%) and 8% (2%, 13%) excess risk for peripheral artery disease, 26% (10%, 41%) and 16% (5%, 27%) for myocardial infarction, 34% (5%, 64%) and 34% (12%, 55%) for ischaemic stroke, and 24% (12%, 36%) and 18% (10%, 27%) for any ASCVD, respectively (Fig. 4). In contrast, elevated LDL-cholesterol did not explain excess risk. Overall, results were similar in statin non-users and in non-smokers (ESM Fig. 16), while statistical power was too low for reliable estimates for statin users and smokers.

Individuals with diabetes and non-fasting plasma glucose ≥6.5 mmol/l had higher excess risk of ASCVD (ESM Fig. 17) and higher levels of remnant cholesterol (ESM Fig. 18) compared to individuals with diabetes and non-fasting plasma glucose <6.5 mmol/l. Remnant cholesterol explained excess risk of ASCVD conferred by diabetes with non-fasting plasma glucose ≥6.5 mmol/l (ESM Fig. 19), while it could not be determined if remnant cholesterol explains excess risk conferred by diabetes with non-fasting plasma glucose <6.5 mmol/l; however, this analysis had lower power due to the lower excess risk of ASCVD in these individuals (ESM Fig. 17).

In all individuals, *p* for interaction for the risk of peripheral artery disease was 0.58 between diabetes vs no diabetes and remnant cholesterol levels, and 0.21 between diabetes vs no diabetes and LDL-cholesterol levels. Corresponding values were 0.57 and 0.01 for myocardial infarction, 0.29 and 0.72 for ischaemic stroke, and 0.91 and 0.001 for any ASCVD. In statin non-users, corresponding values were 0.85 and 0.64 for peripheral artery disease, 0.53 and 0.01 for myocardial infarction, 0.52 and 0.51 for ischaemic stroke, and 0.62 and 0.09 for any ASCVD. In statin users, corresponding values were 0.18 and 0.28 for peripheral artery disease, 0.61 and 0.30 for myocardial infarction, 0.58 and 0.92 for ischaemic stroke, and 0.86 and 0.68 for any ASCVD.

## Discussion

In this contemporary cohort study of 4569 individuals with diabetes followed for a median of 8 years, a doubling of remnant cholesterol was associated with 1.6-fold increased risk of any ASCVD, including 1.6-fold risk of peripheral artery disease, 1.8-fold risk of myocardial infarction, and 1.5-fold risk of ischaemic stroke. Furthermore, elevated remnant cholesterol and low-grade inflammation, but not elevated LDL-cholesterol, explained substantial fractions of the excess risk of ASCVD conferred by diabetes.

### Potential explanations for our findings

Individuals with diabetes commonly have elevated remnant cholesterol levels, probably as a result of both increased production of triglyceride-rich lipoproteins due to dietary and lifestyle factors, and increased retention of triglyceride-rich lipoproteins in plasma due to insulin resistance and hyperglycaemia, which inhibit lipoprotein lipase activity [8, 15]. Remnant cholesterol in triglyceride-rich lipoproteins can penetrate the arterial intima and accumulate in atherosclerotic plaques alongside LDL [3, 16], and elevated remnant cholesterol is a causal risk factor for ASCVD [2, 3]. Indeed, most cases of peripheral artery disease and myocardial infarction result from atherosclerosis [17], as do many cases of ischaemic stroke [18]. Additionally, our results in individuals with high non-fasting plasma glucose indicate that elevated remnant cholesterol may explain increased ASCVD risk conferred by postprandial lipaemia or poor glycaemic control [19]. This may be because postprandial lipaemia better reflects cumulative exposure to high levels of triglyceride-rich lipoproteins compared with fasting lipaemia, as most of the day is spent in a postprandial state [20], or because postprandial lipaemia reflects adverse lifestyle factors including poor diet, alcohol consumption and smoking [21].

Another suggested atherogenic driver of diabetic dyslipidaemia is elevated small dense LDL, leading to a higher number of apolipoprotein B-containing lipoproteins [22]. However, our results adjusted for apolipoprotein B suggest that only the association between elevated remnant cholesterol and increased risk of peripheral artery disease could be explained by a higher particle number. Recently, LDL triglycerides, which are highly correlated with total plasma triglycerides and remnant cholesterol, have also gained interest due to a strong association with risk of ASCVD [23, 24]. However, since elevated LDL triglycerides have no known role in the development of atherosclerosis mechanistically, it may simply be an efficient marker of elevated remnant cholesterol. Remnant cholesterol was calculated from plasma triglycerides in most individuals and is therefore highly correlated with plasma triglycerides [2]. However, as remnant cholesterol measured directly using NMR spectroscopy in a subset of the cohort had similar associations with risk of ASCVD, and as cholesterol, not triglycerides, accumulates in the atherosclerotic plaque, we suggest that our results are explained by the cholesterol content of triglyceride-rich lipoproteins, and not the triglyceride content [3].

In our study, elevated remnant cholesterol as well as low-grade inflammation were common in individuals with diabetes and were associated with increased risk of ASCVD in individuals with diabetes. Summarising these findings, elevated remnant cholesterol explained 24% and low-grade inflammation explained 18% of the excess risk of any ASCVD in diabetes, which supports the hypothesis that elevated remnant cholesterol and low-grade inflammation may be two of several mechanisms through which diabetes increases risk of ASCVD [2, 8, 25, 26]. Although it has been documented that elevated remnant cholesterol is a causal driver of whole-body low-grade inflammation in individuals in the general population [27, 28], the mechanistic links between elevated remnant cholesterol, low-grade inflammation and poor glycaemic control in the context of ASCVD in diabetes are directions for future study.

Meanwhile, elevated LDL-cholesterol was not associated with increased risk of ASCVD in this contemporary Danish diabetes population, likely to be in part because 60% of individuals were on statins. It is also possible that individuals without statin use at baseline but with high levels of LDL-cholesterol were aggressively treated with statins during follow-up; indeed, the prevalence of lipid-lowering therapy use in Denmark has increased from 35 to 115 per 1000 inhabitants between the start of the Copenhagen General Population Study in 2003 and the end of follow-up in 2018 (http://www.medstat.dk, accessed 20 January 2023). Additionally, it is likely that some individuals with statin use at baseline discontinued their treatment during follow-up. Because of changes in the use of statins and other medications such as glucose-lowering medication during follow-up, baseline cholesterol levels may not reflect cumulative cholesterol exposure, which would bias our results towards lower estimates. Contrasting the case for elevated LDL-cholesterol, elevated remnant cholesterol is not a direct indication for statin therapy in clinical guidelines, which means that individuals with high remnant cholesterol at baseline are probably less likely to be prescribed statins during follow-up compared to individuals with high LDL-cholesterol. Additionally, statins have a smaller effect on remnant cholesterol than on LDL-cholesterol, which could also explain why the association between elevated remnant cholesterol and risk of ASCVD was similar regardless of statin use. In summary, the lack of association between LDL-cholesterol and risk of ASCVD may be interpreted as a testimony to the prevalent and intensive treatment of elevated LDL-cholesterol in individuals with diabetes in Denmark, and *not* as a contradiction to evidence from clinical trials showing that lowering LDL-cholesterol from already low levels with ezetimibe [29] and proprotein convertase subtilisin/kexin type 9 inhibitors [30, 31] reduces risk of ASCVD in individuals with diabetes.

### Previous studies

In support of some of our findings, studies of general population cohorts [4–6, 32, 33] corroborate the association between elevated remnant cholesterol and increased risk of peripheral artery disease, myocardial infarction, ischaemic stroke and any ASCVD in individuals with diabetes. Additionally, some studies have found associations between elevated remnant cholesterol and increased risk of coronary artery disease and cardiovascular death in individuals with diabetes and prediabetes [34–36]. Taken together, in the present study we demonstrate in a contemporary population with access to modern primary prevention that elevated remnant cholesterol explained 24% of the excess risk of ASCVD in those with diabetes vs those without diabetes, while LDL-cholesterol did not explain any excess risk of ASCVD.

### Strengths

The relatively large size of this diabetes cohort, long follow-up without losses, and large number of events collected from nationwide registers led to enough statistical power for clinically important stratified analyses. Other strengths include that Danish registry diagnoses of myocardial infarction have high diagnostic accuracy [37] and that ischaemic stroke diagnoses were validated in each patient individually [5]. Further, standard laboratory measurements were used to calculate remnant cholesterol, making the results easily applicable in a clinical setting. Additionally, our study period started at a time when fewer individuals with diabetes received statin treatment than now, allowing us to compare individuals with and without statin treatment at baseline. Finally, it is a strength that the relatively high use of statins during the study means that our results are likely to be generalisable to other contemporary populations in high-income countries.

### Limitations

Observational studies are always limited by potential confounding and reverse causation, which means causal conclusions are difficult to draw. Although we adjusted for important known risk factors, residual and unmeasured confounding cannot be completely excluded. One potential unobserved confounder is changes in statin and glucose-lowering treatment after the baseline examination, as described above. Another limitation is that we could not adjust for reliable markers of glycaemic control (fasting glucose or HbA_1c_) since they were not measured, and therefore we only adjusted for non-fasting glucose, which may reflect postprandial lipaemia, poor glycaemic control or both.

Concerning potential limitations, some may argue that calculated remnant cholesterol just reflects triglycerides; however, calculated remnant cholesterol is strongly correlated with directly measured remnant cholesterol [32, 38]. Additionally, remnant cholesterol measured directly using NMR spectroscopy in a subset of the present cohort showed similar associations with risk of ASCVD. A benefit of calculated remnant cholesterol is that it is inexpensive and can be easily calculated from a standard lipid profile. An additional limitation is the accuracy of peripheral artery disease diagnoses, which has previously been found to be moderate [39]. However, such diagnostic inaccuracy, if non-differential for levels of remnant cholesterol, will only bias our results for peripheral artery disease and ASCVD towards the null hypothesis and therefore cannot explain the association between elevated remnant cholesterol and increased risk of ASCVD. Lastly, this study was carried out in a homogeneous cohort of exclusively northern European ethnicity, which may limit the generalisability of the results. However, we are not aware of data indicating that atherogenicity of remnant cholesterol would differ by ethnicity.

### Clinical context and future studies

We observed that individuals with diabetes still have high excess risk of ASCVD, especially peripheral artery disease, despite access to statins, glucose-lowering drugs and other medications. Importantly, our results do not contradict the need of achieving optimal control of LDL-cholesterol for prevention of ASCVD in individuals with diabetes. However, excess risk of ASCVD in populations with high use of statins could be caused by other factors, including elevated remnant cholesterol and low-grade inflammation.

Indeed, elevated remnant cholesterol may be used as a marker of increased risk of ASCVD in diabetes; importantly, elevated remnant cholesterol may particularly explain risk conferred by diabetes with high non-fasting glucose, which indicates the importance of postprandial lipaemia and glycaemic control for elevated remnant cholesterol and increased ASCVD risk in diabetes [19]. Whether elevated remnant cholesterol should also be used directly as a treatment target remains to be determined. Recently, the Pemafibrate to Reduce Cardiovascular Outcomes by Reducing Triglycerides in Patients With Diabetes Trial (PROMINENT) was terminated early due to futility. However, the decrease in mass of remnant cholesterol by pemafibrate was accompanied by an even larger increase in LDL-cholesterol mass and furthermore by an increase in number of atherogenic lipoproteins assessed as increased apolipoprotein B [40]; in other words, the total atherogenic cholesterol content and total number of atherogenic lipoproteins in plasma were increased in the pemafibrate vs placebo arm of PROMINENT, likely explaining the slight trend towards more ASCVD in the pemafibrate arm of the study. Ongoing clinical trials of apolipoprotein C3 and angiopoietin-like 3 inhibition will provide further valuable information on whether substantial remnant cholesterol-lowering therapy can prevent ASCVD in diabetes, and if there are no adverse effects such as LDL-cholesterol and apolipoprotein B increases, they may provide novel treatment options [41].

### Conclusions

A doubling of remnant cholesterol in individuals with diabetes was associated with a 1.6-fold increased risk of ASCVD, which included 1.6-fold risk of peripheral artery disease, 1.8-fold risk of myocardial infarction and 1.5-fold risk of ischaemic stroke. Elevated remnant cholesterol explained 24% and low-grade inflammation explained 18% of the excess risk of ASCVD in diabetes. Clinical trials should test if substantial remnant cholesterol-lowering therapy without increases in LDL-cholesterol and apolipoprotein B can prevent ASCVD in diabetes.

### Supplementary Information

Below is the link to the electronic supplementary material.

Supplementary file1 (PDF 1002 KB)

## Data Availability

The data cannot be shared due to regulations by the Danish Data Protection Agency. However, additional analyses can be performed upon reasonable request to the corresponding author.

## Abbreviations

ASCVD: Atherosclerotic cardiovascular disease
PROMINENT: Pemafibrate to Reduce Cardiovascular Outcomes by Reducing Triglycerides in Patients With Diabetes
