# Supplementary material for: Elevated remnant cholesterol and atherosclerotic cardiovascular disease in diabetes: a population-based prospective cohort study
Source: Diabetologia. 2023 Sep 30;66(12):2238–49. doi: 10.1007/s00125-023-06016-0 (PMC10627991; doi:10.1007/s00125-023-06016-0)
Supplement: Supplementary file 1 — Supplementary file1 (PDF 1002 KB) [file 125_2023_6016_MOESM1_ESM.pdf]

ELECTRONIC SUPPLEMENTARY MATERIAL (ESM)

Elevated remnant cholesterol and atherosclerotic cardiovascular disease in diabetes: a population-based prospective cohort study

Benjamin N. Wadström[a,-c], MD, Kasper M. Pedersen[a-c], MD, Anders B. Wulff[a-c], MD, Børge G. Nordestgaard[a-c], DMSc

[a]Department of Clinical Biochemistry, Herlev and Gentofte Hospital, Copenhagen University Hospital, Borgmester Ib Juuls Vej 73, entrance 7, 4th floor, N5, DK-2730 Herlev, Denmark.

[b]The Copenhagen General Population Study, Herlev and Gentofte Hospital, Copenhagen University Hospital, Borgmester Ib Juuls Vej 73 entrance 7, 4th floor, M3, Herlev DK-2730, Denmark.

[c]Department of Clinical Medicine, Faculty of Health and Medical Sciences, University of Copenhagen, Blegdamsvej 3b 33.5., Copenhagen DK-2200, Denmark.

Address for correspondence: Børge G. Nordestgaard, Professor, Chief Physician, MD, DMSc

Borgmester Ib Juuls Vej 73, entrance 7, 4th floor, N5, Department of Clinical Biochemistry, Herlev og Gentofte Hospital, Copenhagen University Hospital, DK-2730 Herlev, Denmark

Phone: +45 3868 3297

E-mail: boerge.nordestgaard@regionh.dk

Contents

ESM Methods 3

ESM References 4

ESM Tables....5

ESM Table 1 5
ESM Table 2 6
ESM Table 3 7

ESM Figures 8

ESM Fig. 1. 8
ESM Fig. 2 9
ESM Fig. 3 10
ESM Fig. 4 11
ESM Fig. 5 12
ESM Fig. 6 13
ESM Fig. 7 14
ESM Fig. 8 15
ESM Fig. 9 16
ESM Fig. 10 17
ESM Fig. 11 18
ESM Fig. 12 19
ESM Fig. 13 20
ESM Fig. 14 21
ESM Fig. 15 22
ESM Fig. 16 23
ESM Fig. 17 24
ESM Fig. 18 25
ESM Fig. 19 26

ESM Methods

Age with left truncation as time scale

Individuals were included from age at baseline until age at event or censoring. Survival age as timescale with exclusion of time before the study is known as age timescale with left truncation, entry age-adjusted age-scale, age timescale with delayed entry, or age timescale with entry at baseline age. This approach adjusts for age at baseline in a non-parametric way (i.e. does not require statistical modelling of the relationship between age and outcome), and as such minimizes risk of bias due to age(1).

To illustrate this, a simulation study by Pencina et. al (in which the entry-age-adjusted age-scale model was considered "the most appropriate" for epidemiological studies) concluded that an incorrect functional form of the age term in time-on-study models may lead to inflated or deflated probabilities of survival and inferior calibration(2). Despite this, they found that regression coefficients for risk factors of interest were very similar in other commonly used models using time-on-study as time scale with parametric adjustment for entry age. As such, age timescale with left truncation and time-on-study timescale with parametric adjustment for age perform equally well for estimation of coefficients, which are used to yield relative risks such as hazard ratios. Cox regressions were additionally adjusted for birth year to adjust for calendar effects (e.g. changes in diagnostics and treatment practices over time).

Sensitivity analysis

In sensitivity analyses, we examined the associations of elevated remnant cholesterol and low-density lipoprotein (LDL) cholesterol with risk of atherosclerotic cardiovascular disease (ASCVD) i) with additional adjustment for body mass index and ASCVD before baseline, ii) with Fine and Gray competing risk regression with death as competing event, iii) using time on study as timescale with adjustment for age using restricted cubic splines, iv) without correction for regression dilution bias, v) additionally adjusting for high-sensitivity C-reactive protein, glucose-lowering medication use, body mass index, apolipoprotein B, high-density lipoprotein (HDL) cholesterol, triglycerides, and LDL triglycerides, vi) using remnant cholesterol and LDL cholesterol calculated from the Martin-Hopkins(3) and the Sampson-NIH(4) formulas, vii) using remnant cholesterol, LDL cholesterol, and LDL triglycerides directly measured using nuclear magnetic resonance spectroscopy, viii) excluding individuals with diabetes type 1, ix) excluding all individuals with ASCVD before baseline, x) stratified by non-fasting plasma glucose, and xi) stratified by sex.

Analysis of explained excess risk

The method used for analysis of explained excess risk is often referred to as mediation analysis. The CMAverse package integrates exposure*mediator interaction in mediation analyses(5). As the association of the mediator with risk of ASCVD may be different in individuals with and without diabetes, interaction is essential to include in analysis of explained excess risk/mediation analysis. This can help estimate the fraction of excess risk of ASCVD in diabetes which may be explained by the explanatory factor/mediator, here termed explained excess risk. Measurement error in remnant cholesterol and C-reactive protein was corrected based on the standard errors from replicate measurements 10 years apart from the Copenhagen City Heart Study (see following section), using quadratic simulation extrapolation with 100 replications(6).

Measurement error correction

Random measurement error biases regression coefficients towards lower estimates (=regression dilution bias). Correction for measurement error was done for remnant cholesterol and LDL cholesterol in all regression analyses using regression calibration(7), except for explained excess risk analyses. Hazard ratios and 95% confidence intervals were corrected for measurement error using repeat measurements from 2,912 individuals without lipid-lowering therapy participating in both the 1991–1994 and 2001–2003 examinations of the Copenhagen City Heart Study(8). Regression calibration, using the same adjustments as in the Cox regressions, was used to yield calibration factors(9). Calibration factors for remnant cholesterol, LDL cholesterol, and high-sensitivity C-reactive protein were 0.48, 0.58, and 0.57. The hazard ratios from the age and sex adjusted model and the multivariable adjusted model, respectively, were corrected by dividing the natural logarithm of the hazard ratio by the corresponding calibration factor.

ESM References

1. Cologne J, Hsu WL, Abbott RD et al. (2012) Proportional hazards regression in epidemiologic follow-up studies: an intuitive consideration of primary time scale. Epidemiology 23(4):565-73. 10.1097/EDE.0b013e318253e418
2. Pencina MJ, Larson MG, D'Agostino RB (2007) Choice of time scale and its effect on significance of predictors in longitudinal studies. Stat Med 26(6):1343-59. 10.1002/sim.2699
3. Martin SS, Blaha MJ, Elshazly MB et al. (2013) Comparison of a novel method vs the Friedewald equation for estimating low-density lipoprotein cholesterol levels from the standard lipid profile. JAMA 310(19):2061-8. 10.1001/jama.2013.280532
4. Sampson M, Ling C, Sun Q et al. (2020) A New Equation for Calculation of Low-Density Lipoprotein Cholesterol in Patients With Normolipidemia and/or Hypertriglyceridemia. JAMA Cardiol 5(5):540-548. 10.1001/jamacardio.2020.0013
5. Shi B, Choirat C, Coull BA, VanderWeele TJ, Valeri L (2021) CMAverse: A Suite of Functions for Reproducible Causal Mediation Analyses. Epidemiology 32(5):e20-e22. 10.1097/EDE.0000000000001378
6. Cook JR, Stefanski LA (1994) Simulation-Extrapolation Estimation in Parametric Measurement Error Models. Journal of the American Statistical Association 89(428):1314-1328. 10.1080/01621459.1994.10476871
7. Rosner B, Spiegelman D, Willett WC (1992) Correction of logistic regression relative risk estimates and confidence intervals for random within-person measurement error. Am J Epidemiol 136(11):1400-13. 10.1093/oxfordjournals.aje.a116453
8. Varbo A, Freiberg JJ, Nordestgaard BG (2015) Extreme nonfasting remnant cholesterol vs extreme LDL cholesterol as contributors to cardiovascular disease and all-cause mortality in 90000 individuals from the general population. Clin Chem 61(3):533-43. 10.1373/clinchem.2014.234146
9. Rosner B, Spiegelman D, Willett WC (1990) Correction of logistic regression relative risk estimates and confidence intervals for measurement error: the case of multiple covariates measured with error. Am J Epidemiol 132(4):734-45. 10.1093/oxfordjournals.aje.a115715

ESM Tables

ESM Table 1. Baseline characteristics of individuals with diabetes in the Copenhagen General Population Study by statin treatment.

	Statin use		
	**Yes**	**No**	**All**
Number of individuals	2,643	1,926	4,569
Men	1,560 (59%)	1,072 (56%)	2,632 (58%)
Age, years	68 (61-73)	65 (56-73)	67 (60-73)
Remnant cholesterol, mmol/L	0.8 (0.5-1.1)	0.8 (0.5-1.2)	0.8 (0.5-1.2)
Remnant cholesterol, mg/dL	31 (21-44)	31 (20-47)	31 (21-45)
LDL cholesterol, mmol/L	2.0 (1.6-2.5)	3.0 (2.4-3.6)	2.3 (1.8-3.1)
LDL cholesterol, mg/dL	77 (62-97)	116 (93-139)	90 (70-120)
Systolic blood pressure, mmHg	145 (133-158)	148 (134-160)	146 (134-160)
Current smokers	412 (16%)	412 (21%)	824 (18%)
Cumulative smoking, pack-years	13 (0-35)	9 (0-31)	11 (0-34)
Non-fasting plasma glucose, mmol/L	6.5 (5.4-8.4)	6.5 (5.3-9.2)	6.5 (5.4-8.6)
Non-fasting plasma glucose, mg/dL	117 (97-151)	117 (95-166)	117 (97-155)
C-reactive protein, mg/L	1.6 (1-2.9)	2.0 (1.2-4.2)	1.7 (1.1-3.3)
Within biological pathway			
Body mass index, kg/m^2	29 (26-32)	28 (25-32)	29 (26-32)
ASCVD	499 (19%)	106 (6%)	928 (20%)
Hypertension	2,365 (89%)	1,540 (80%)	2,489 (84%)
Triglycerides, mmol/L	1.8 (1.2-2.6)	1.8 (1.2-2.8)	1.8 (1.2-2.7)
Triglycerides, mg/dL	159 (108-231)	157 (104-245)	159 (106-238)
Only insulin use	307 (12%)	279 (14%)	586 (13%)
Only other glucose-lowering medication use	1,442 (55%)	709 (37%)	2,151 (47%)
Both insulin and other glucose-lowering medication use	294 (11%)	87 (5%)	381 (8%)
Diabetes characteristics			
Diabetes type 1 diagnosis	431 (16%)	347 (18%)	778 (17%)
Diabetes type 2 diagnosis	1,432 (54%)	747 (39%)	2,179 (48%)
Self-reported diabetes	2,458 (93%)	1,524 (80%)	3,982 (88%)
Non-fasting plasma glucose >11 mmol/L (198 mg/dL)	271 (10%)	344 (18%)	615 (13%)

Values are median (inter-quartile range) for continuous variables and number (percentage) for categorical variables. Statin use indicates use of lipid-lowering medications, which was mostly statins. ASCVD=Atherosclerotic cardiovascular disease, comprising peripheral artery disease, myocardial infarction, and ischaemic stroke before baseline, LDL=Low-density lipoprotein

ESM Table 2. Baseline characteristics of individuals with diabetes in the Copenhagen General Population Study by current smoking.

	Current smoking		
	**Yes**	**No**	**All**
Number of individuals	824	3,745	4,569
Men	476 (58%)	2,156 (58%)	2,632 (58%)
Age, years	63 (55-69)	67 (60-74)	67 (60-73)
Remnant cholesterol, mmol/L	0.9 (0.6-1.3)	0.8 (0.5-1.1)	0.8 (0.5-1.2)
Remnant cholesterol, mg/dL	33 (22-50)	31 (21-44)	31 (21-45)
LDL cholesterol, mmol/L	2.5 (1.9-3.3)	2.3 (1.8-3)	2.3 (1.8-3.1)
LDL cholesterol, mg/dL	97 (73-128)	89 (69-116)	90 (70-120)
Systolic blood pressure, mmHg	142 (130-156)	147 (134-160)	146 (134-160)
Cumulative smoking, pack-years	36 (21-50)	5 (0-27)	11 (0-34)
Non-fasting plasma glucose, mmol/L	6.4 (5-8.4)	6.6 (5.4-8.7)	6.5 (5.4-8.6)
Non-fasting plasma glucose, mg/dL	115 (90-150)	119 (97-157)	117 (97-155)
C-reactive protein, mg/L	2.0 (1.3-4.1)	1.7 (1.1-3.2)	1.7 (1.1-3.3)
Within biological pathway			
Statin use	412 (50%)	2,231 (60%)	2,643 (58%)
Body mass index, kg/m^2	28 (25-31)	29 (26-32)	29 (26-32)
ASCVD	130 (16%)	475 (13%)	605 (13%)
Hypertension	660 (80%)	3,245 (87%)	3,905 (85%)
Triglycerides, mmol/L	1.9 (1.2-3.0)	1.8 (1.2-2.6)	1.8 (1.2-2.7)
Triglycerides, mg/dL	167 (109-269)	156 (105-230)	159 (106-238)
Only insulin use	118 (14%)	468 (12%)	586 (13%)
Only other glucose-lowering medication use	369 (45%)	1,782 (48%)	2,151 (47%)
Both insulin and other glucose-lowering medication use	44 (5%)	337 (9%)	381 (8%)
Diabetes characteristics			
Diabetes type 1 diagnosis	149 (18%)	629 (17%)	778 (17%)
Diabetes type 2 diagnosis	382 (46%)	1,797 (48%)	2,179 (48%)
Self-reported diabetes	707 (86%)	3,275 (88%)	3,982 (88%)
Non-fasting plasma glucose >11 mmol/L (198 mg/dL)	119 (14%)	496 (13%)	615 (13%)

Values are median (inter-quartile range) for continuous variables and number (percentage) for categorical variables. Statin use indicates use of lipid-lowering medications, which was mostly statins. ASCVD=Atherosclerotic cardiovascular disease, comprising peripheral artery disease, myocardial infarction, and ischaemic stroke before baseline, LDL=Low-density lipoprotein

ESM Table 3. Baseline characteristics of individuals with diabetes in the Copenhagen General Population Study by diabetes type.

	Diabetes type			
	**Type 2**	**Type 1**	**Type ambiguity**	**All**
Number of individuals	3,791	246	532	4,569
Men	2,179 (57%)	130 (53%)	323 (61%)	2,632 (58%)
Age, years	67 (61-74)	51 (45-56)	68 (62-73)	67 (60-73)
Remnant cholesterol, mmol/L	0.8 (0.6-1.2)	0.4 (0.3-0.8)	0.7 (0.4-1.0)	0.8 (0.5-1.2)
Remnant cholesterol, mg/dL	32 (22-47)	17 (13-29)	26 (17-40)	31 (21-45)
LDL cholesterol, mmol/L	2.4 (1.8-3.1)	2.5 (2.0-2.9)	2.2 (1.6-2.8)	2.3 (1.8-3.1)
LDL cholesterol, mg/dL	92 (70-120)	95 (77-112)	85 (64-109)	90 (70-120)
Systolic blood pressure, mmHg	147 (134-160)	138 (126-151)	145 (132-157)	146 (134-160)
Current smokers	675 (18%)	58 (24%)	91 (17%)	824 (18%)
Cumulative smoking, pack-years	12 (0-34)	3 (0-20)	10 (0-34)	11 (0-34)
Non-fasting plasma glucose, mmol/L	6.4 (5.4-8.2)	8.8 (5.9-12.7)	7.3 (5.5-10.7)	6.5 (5.4-8.6)
Non-fasting plasma glucose, mg/dL	115 (97-148)	159 (106-229)	131 (99-193)	117 (97-155)
C-reactive protein, mg/L	1.8 (1.1-3.5)	1.3 (0.8-2.5)	1.7 (1.1-2.9)	1.7 (1.1-3.3)
Within biological pathway				
Statin use	2,212 (58%)	95 (39%)	336 (63%)	2,643 (58%)
Body mass index, kg/m^2	29 (26-32)	25 (23-28)	28 (24-32)	29 (26-32)
ASCVD	477 (13%)	12 (5%)	116 (22%)	605 (13%)
Hypertension	3,265 (86%)	164 (67%)	476 (89%)	3,905 (85%)
Triglycerides, mmol/L	1.9 (1.3-2.8)	1.0 (0.7-1.7)	1.5 (1.0-2.3)	1.8 (1.2-2.7)
Triglycerides, mg/dL	167 (114-244)	90 (66-148)	134 (85-205)	159 (106-238)
Only insulin use	111 (3%)	207 (84%)	268 (50%)	586 (13%)
Only other glucose-lowering medication use	2,077 (55%)	8 (3%)	66 (12%)	2,151 (47%)
Both insulin and other glucose-lowering medication use	253 (7%)	18 (7%)	110 (21%)	381 (8%)
Diabetes characteristics				
Diabetes type 1 diagnosis	0 (0%)	246 (100%)	532 (100%)	778 (17%)
Diabetes type 2 diagnosis	1,659 (44%)	171 (70%)	349 (66%)	2,179 (48%)
Self-reported diabetes	3,289 (87%)	237 (97%)	456 (86%)	3,982 (88%)
Non-fasting plasma glucose >11 mmol/L (198 mg/dL)	411 (11%)	86 (35%)	118 (22%)	615 (13%)

Values are median (inter-quartile range) for continuous variables and number (percentage) for categorical variables. Statin use indicates use of lipid-lowering medications, which was mostly statins. ASCVD=Atherosclerotic cardiovascular disease, comprising peripheral artery disease, myocardial infarction, and ischaemic stroke before baseline, LDL=Low-density lipoprotein

ESM Figures

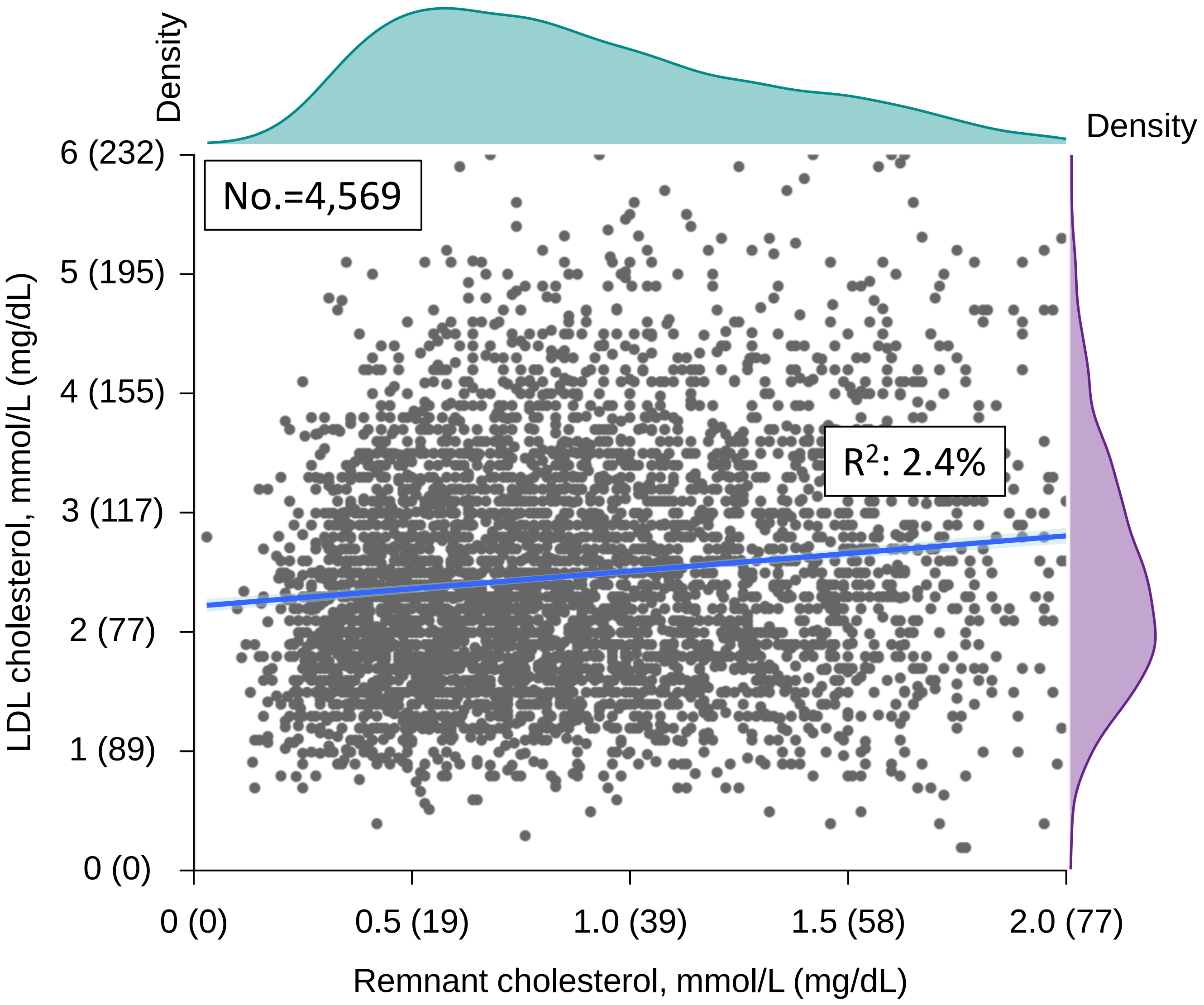

ESM Fig. 1. Correlation between remnant cholesterol and LDL cholesterol in individuals with diabetes from the Copenhagen General Population Study.

The blue line is linear regression. R^2 is the coefficient of determination in percent. LDL=Low-density lipoprotein. No.=Number

Group	Endpoint		No. of individuals	No. of events	No. of events per 1,000 person-years (95% CI): At median level	At double median level	Hazard ratio (95% CI) per double remnant or LDL cholesterol, multivariable adjusted	*p*
No statins	Peripheral artery disease		1895	84				
		Remnant cholesterol			5 (4, 6)	7 (5, 9)	1.5 (0.9, 2.8)	0.15
		LDL cholesterol			5 (3, 6)	6 (4, 8)	1.7 (0.7, 3.9)	0.21
	Myocardial infarction		1878	97				
		Remnant cholesterol			6 (5, 7)	8 (6, 10)	1.7 (1.0, 3.0)	0.058
		LDL cholesterol			6 (5, 8)	6 (4, 8)	1.0 (0.5, 2.0)	0.92
	Ischemic stroke		1831	105				
		Remnant cholesterol			7 (5, 8)	8 (6, 11)	1.3 (0.8, 2.3)	0.31
		LDL cholesterol			6 (5, 8)	8 (6, 11)	1.4 (0.7, 2.9)	0.37
	Any ASCVD		1763	226				
		Remnant cholesterol			15 (13, 17)	20 (17, 24)	1.4 (1.0, 2.0)	0.068
		LDL cholesterol			15 (13, 18)	16 (13, 20)	1.2 (0.7, 1.9)	0.57
Non-smokers	Peripheral artery disease		3631	153				
		Remnant cholesterol			5 (4, 6)	7 (6, 9)	2.3 (1.4, 3.7)	0.00049
		LDL cholesterol			5 (4, 6)	4 (3, 6)	1.0 (0.6, 1.5)	0.88
	Myocardial infarction		3477	187				
		Remnant cholesterol			6 (6, 7)	9 (7, 11)	1.8 (1.2, 2.7)	0.0038
		LDL cholesterol			7 (6, 8)	6 (4, 8)	1.0 (0.7, 1.5)	0.98
	Ischemic stroke		3443	170				
		Remnant cholesterol			6 (5, 7)	7 (6, 9)	1.4 (0.9, 2.1)	0.16
		LDL cholesterol			6 (5, 7)	6 (5, 8)	1.1 (0.7, 1.7)	0.68
	Any ASCVD		3136	370				
		Remnant cholesterol			15 (13, 16)	20 (17, 23)	1.7 (1.2, 2.2)	0.00071
		LDL cholesterol			15 (13, 16)	13 (11, 16)	0.9 (0.7, 1.2)	0.67

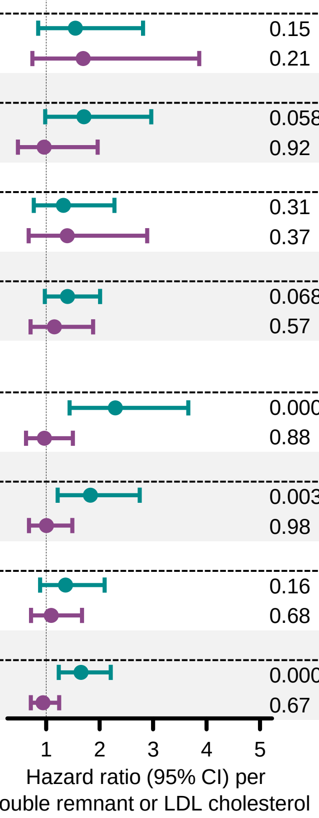

ESM Fig. 2. **Risk of ASCVD per doubling of remnant cholesterol and LDL cholesterol in individuals with diabetes <u>who were not using statins and who were non-smokers</u>, respectively, from the Copenhagen General Population Study**

Cox regression multivariable adjusted for age, sex, systolic blood pressure, diastolic blood pressure, smoking status (except for analyses in non-smokers), cumulative smoking, birth year, non-fasting plasma glucose, and LDL cholesterol (in remnant cholesterol analyses) or remnant cholesterol (in LDL cholesterol analyses). ASCVD=Atherosclerotic cardiovascular disease, CI=Confidence interval, LDL=Low-density lipoprotein, No.=Number.

	Endpoint		No. of individuals	No. of events	No. of events per 1,000 person-years (95% CI): At median level	No. of events per 1,000 person-years (95% CI): At double median level	Hazard ratio (95% CI) per double remnant or LDL cholesterol, multivariable adjusted	*p*
Statin users	Peripheral artery disease		2519	152				
		Remnant cholesterol			7 (6, 9)	9 (7, 12)	1.6 (1.0, 2.5)	0.064
		LDL cholesterol			7 (6, 9)	6 (4, 9)	1.0 (0.6, 1.6)	0.98
	Myocardial infarction		2359	137				
		Remnant cholesterol			7 (6, 9)	9 (7, 12)	2.0 (1.2, 3.2)	0.0074
		LDL cholesterol			7 (6, 9)	8 (5, 12)	1.3 (0.8, 2.3)	0.27
	Ischemic stroke		2371	121				
		Remnant cholesterol			6 (5, 8)	8 (6, 10)	1.5 (0.9, 2.6)	0.10
		LDL cholesterol			6 (5, 8)	6 (4, 9)	1.1 (0.7, 1.9)	0.71
	Any ASCVD		2043	272				
		Remnant cholesterol			17 (15, 19)	22 (18, 27)	1.7 (1.2, 2.5)	0.0015
		LDL cholesterol			16 (14, 19)	13 (10, 18)	0.9 (0.6, 1.2)	0.41
Smokers	Peripheral artery disease		783	83				
		Remnant cholesterol			12 (10, 15)	12 (9, 17)	0.9 (0.5, 1.6)	0.71
		LDL cholesterol			12 (10, 15)	9 (6, 13)	0.8 (0.4, 1.3)	0.33
	Myocardial infarction		760	47				
		Remnant cholesterol			7 (5, 9)	9 (6, 13)	1.7 (0.8, 3.9)	0.19
		LDL cholesterol			7 (5, 9)	7 (4, 12)	1.0 (0.4, 2.4)	0.95
	Ischemic stroke		759	56				
		Remnant cholesterol			8 (6, 11)	11 (8, 15)	1.9 (0.9, 3.9)	0.10
		LDL cholesterol			9 (7, 11)	10 (6, 15)	1.2 (0.6, 2.6)	0.59
	Any ASCVD		670	128				
		Remnant cholesterol			23 (19, 27)	27 (21, 34)	1.4 (0.9, 2.3)	0.14
		LDL cholesterol			24 (20, 29)	19 (14, 26)	0.7 (0.4, 1.2)	0.20

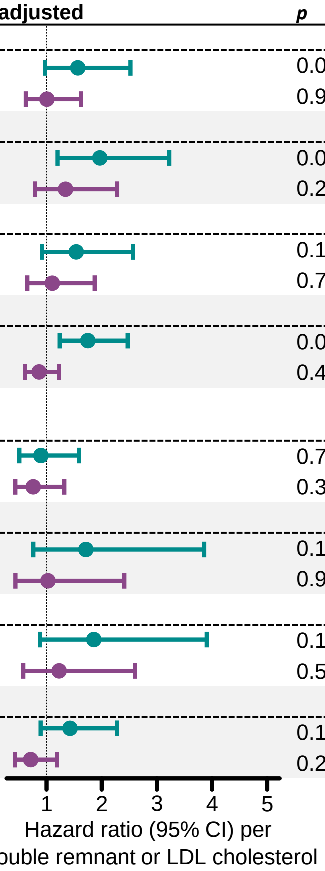

ESM Fig. 3. Risk of ASCVD per doubling of remnant cholesterol and LDL cholesterol in individuals with diabetes <u>who were using statins and who were smokers</u>, respectively, from the Copenhagen General Population Study.

Cox regression adjusted for age, sex, systolic blood pressure, diastolic blood pressure, smoking status (except for analyses in smokers), cumulative smoking, birth year, non-fasting plasma glucose, and LDL cholesterol (in remnant cholesterol analyses) or remnant cholesterol (in LDL cholesterol analyses). ASCVD=Atherosclerotic cardiovascular disease, CI=Confidence interval, LDL=Low-density lipoprotein, No.=Number.

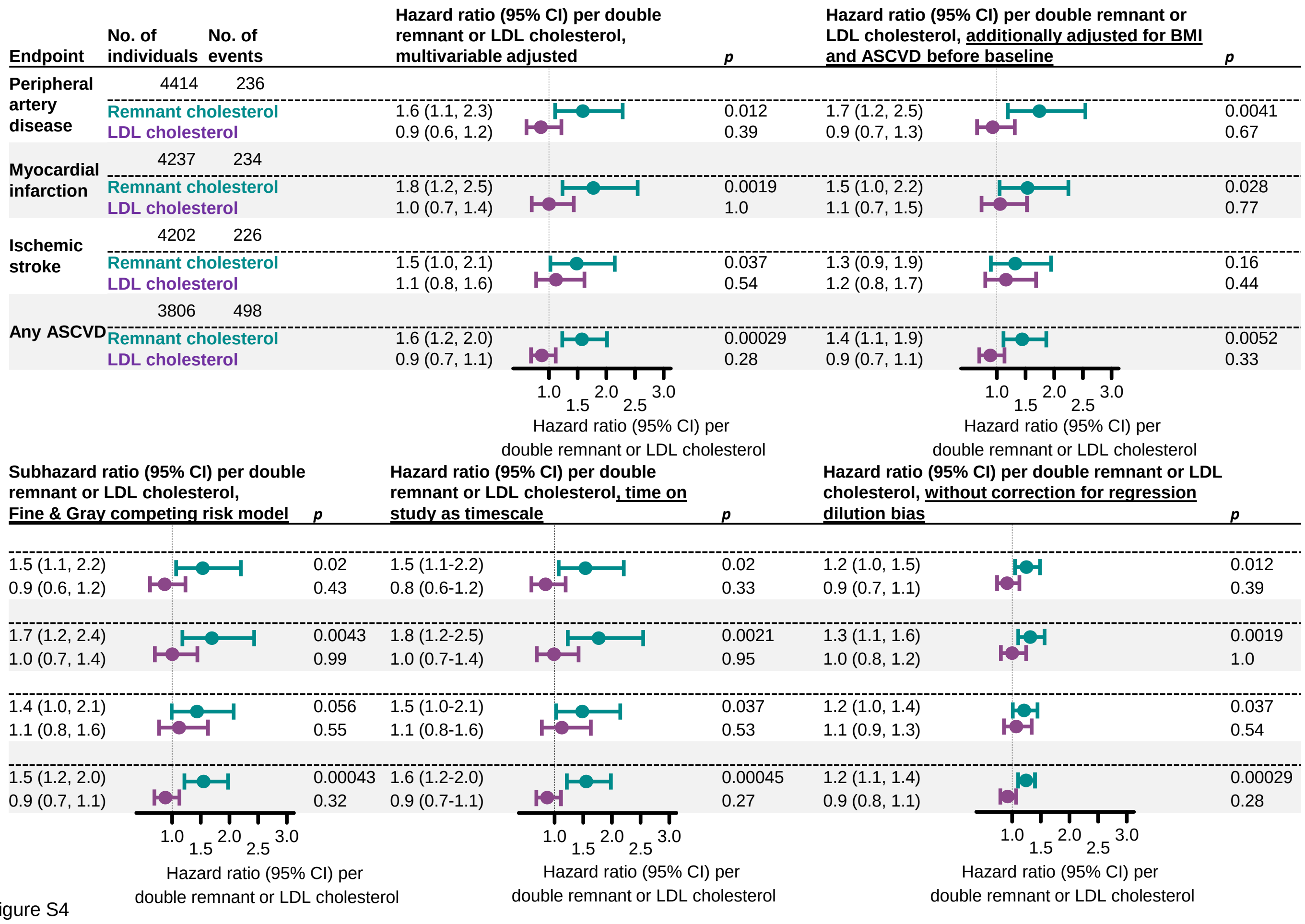

Figure S4

ESM Fig. 4. Sensitivity analyses of risk of ASCVD per doubling of remnant cholesterol and LDL cholesterol in individuals with diabetes from the Copenhagen General Population Study.

Results from i) original multivariable adjusted Cox model, ii) additionally adjusted for BMI and ASCVD before baseline, iii) Fine and Gray competing risk regression with death as competing event, iv) using time on study as timescale, and v) without correction for regression dilution bias. The original Cox model was adjusted for age, sex, systolic blood pressure, diastolic blood pressure, smoking status, cumulative smoking, birth year, non-fasting plasma glucose, and LDL cholesterol (in remnant cholesterol analyses) or remnant cholesterol (in LDL cholesterol analyses). ASCVD=Atherosclerotic cardiovascular disease, BMI=Body mass index, CI=Confidence interval LDL=Low-density lipoprotein, No.=Number.

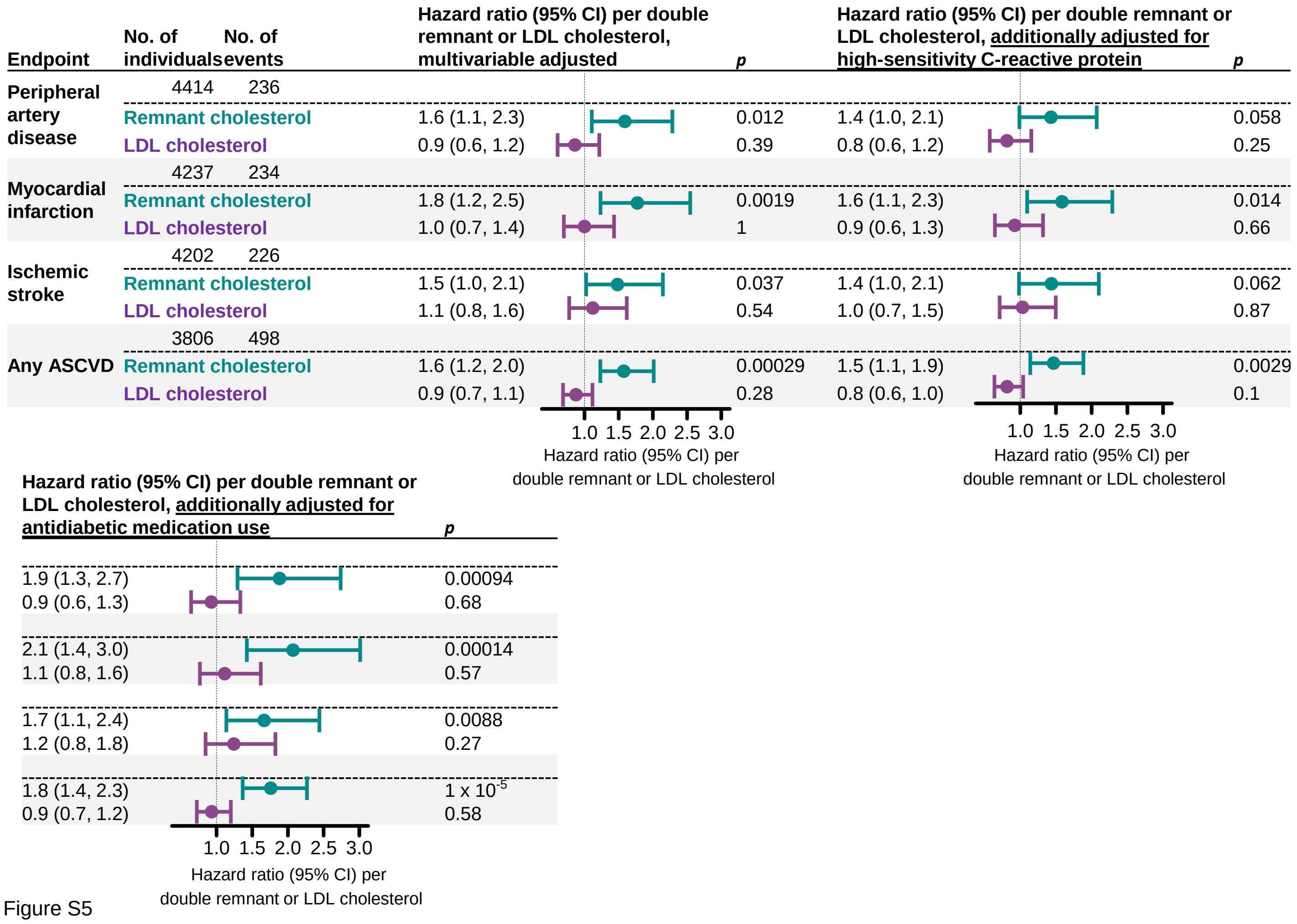

Figure S5

ESM Fig. 5. Risk of ASCVD per doubling of remnant cholesterol and LDL cholesterol in individuals with diabetes from the Copenhagen General Population Study after adjustment for high-sensitivity C-reactive protein and antidiabetic medication use

Results from i) original Cox model, ii) additionally adjusted for high-sensitivity C-reactive protein, iii) additionally adjusted for antidiabetic medication use. The original Cox model was adjusted for age, sex, systolic blood pressure, diastolic blood pressure, smoking status, cumulative smoking, birth year, non-fasting plasma glucose, and LDL cholesterol (in remnant cholesterol analyses) or remnant cholesterol (in LDL cholesterol analyses). Antidiabetic medication use was either no antidiabetic medication, only insulin, only other antidiabetic medication, or both insulin and other antidiabetic medication. ASCVD=Atherosclerotic cardiovascular disease, CI=Confidence interval, HDL=High-density lipoprotein, LDL=Low-density lipoprotein, No.=Number.

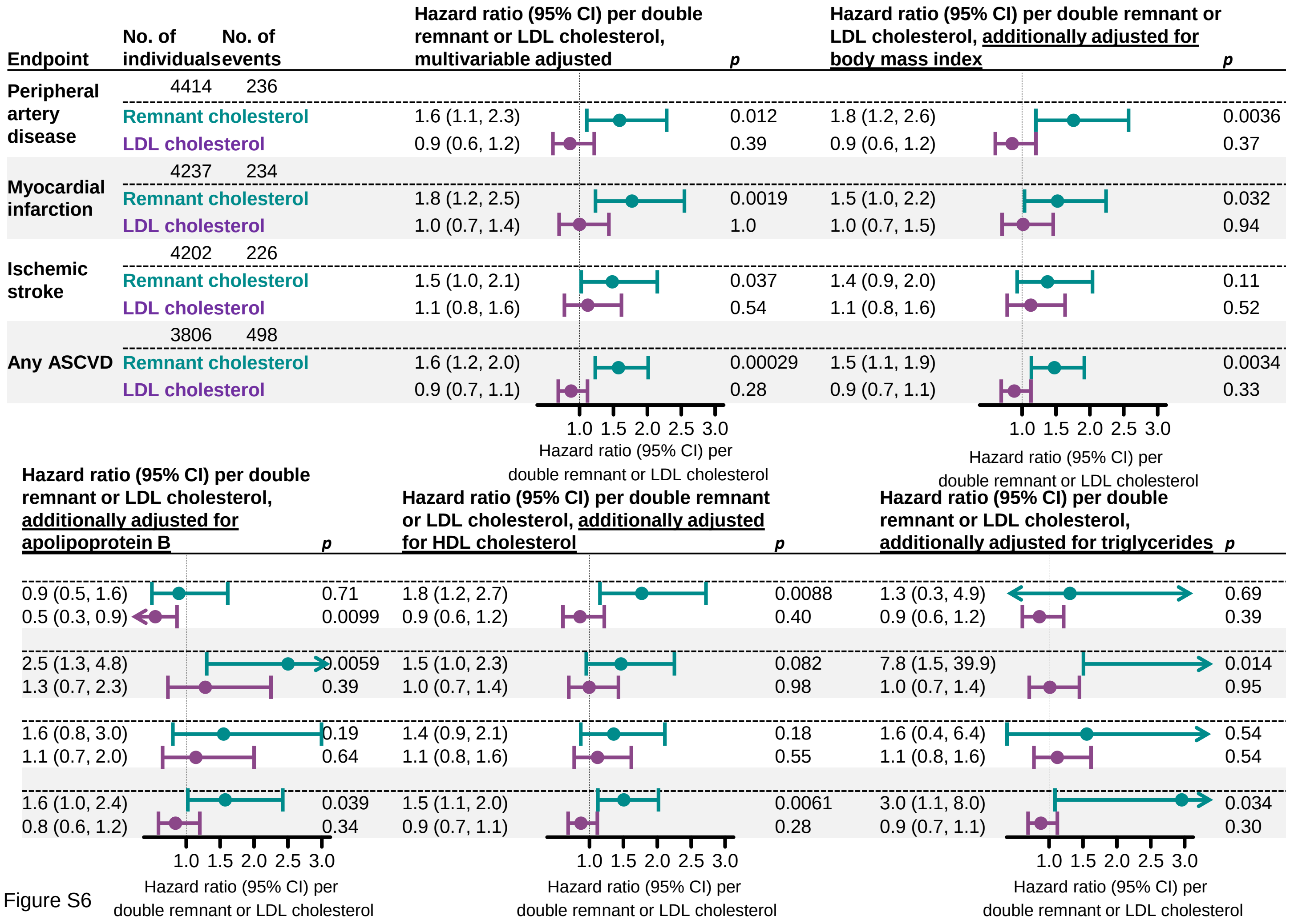

Figure S6

ESM Fig. 6. Risk of ASCVD per doubling of remnant cholesterol and LDL cholesterol in individuals with diabetes from the Copenhagen General Population Study <u>after adjustment for body mass index, apolipoprotein B, HDL cholesterol, and triglycerides.</u>

Results from i) original Cox model, <u>ii) additionally adjusted for body mass index, iii) additionally adjusted for apolipoprotein B, iv) additionally adjusted for HDL cholesterol, and v) additionally adjusted for triglycerides.</u> The original Cox model was adjusted for age, sex, systolic blood pressure, diastolic blood pressure, smoking status, cumulative smoking, birth year, non-fasting plasma glucose, and LDL cholesterol (in remnant cholesterol analyses) or remnant cholesterol (in LDL cholesterol analyses). ASCVD=Atherosclerotic cardiovascular disease, CI=Confidence interval, HDL=High-density lipoprotein, LDL=Low-density lipoprotein, No.=Number.

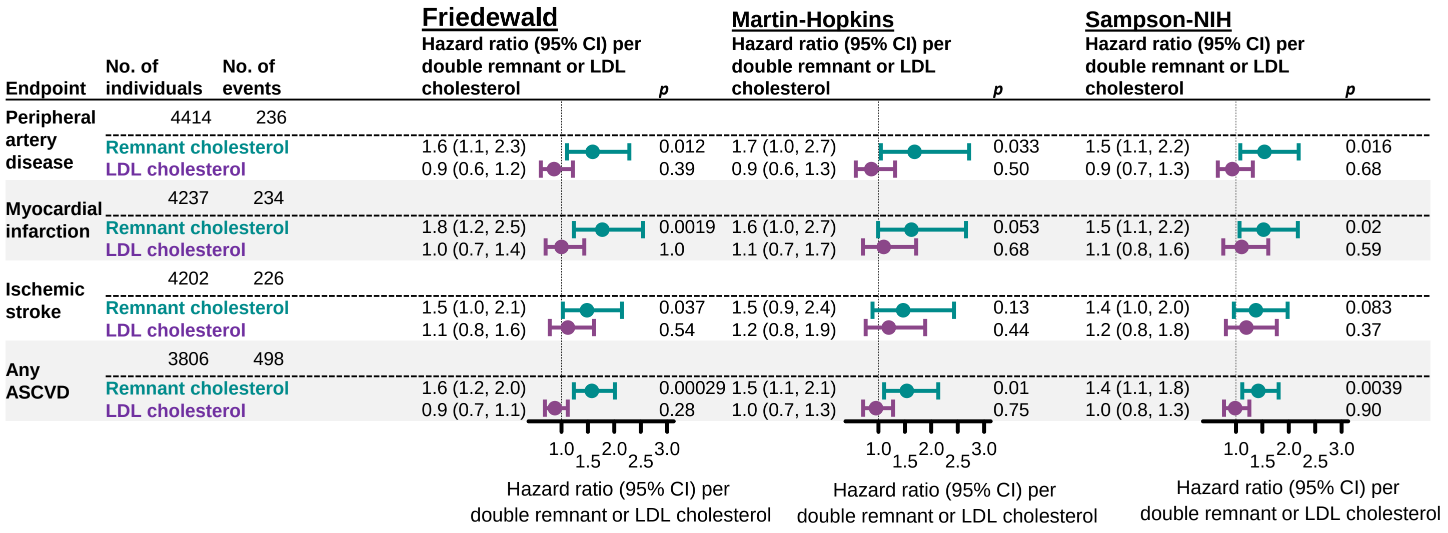

ESM Fig. 7. Risk of ASCVD per doubling of remnant cholesterol and LDL cholesterol in individuals with diabetes from the Copenhagen General Population Study <u>with different formulas for calculating remnant cholesterol and LDL cholesterol.</u>

Results from i) Cox model using the Friedewald formula (original model)<u>, ii) using the Martin-Hopkins formula, and iii) using the Sampson-NIH formula</u>. Adjusted for age, sex, systolic blood pressure, diastolic blood pressure, smoking status, cumulative smoking, birth year, non-fasting plasma glucose, and LDL cholesterol (in remnant cholesterol analyses) or remnant cholesterol (in LDL cholesterol analyses). ASCVD=Atherosclerotic cardiovascular disease, CI=Confidence interval LDL=Low-density lipoprotein, No.=Number.

		No diabetes type 1				No ASCVD before baseline			
**Endpoint**		**No. of individuals**	**No. of events**	**Hazard ratio (95% CI) per double remnant or LDL cholesterol, multivariable adjusted**	***p***	**No. of individuals**	**No. of events**	**Hazard ratio (95% CI) per double remnant or LDL cholesterol, multivariable adjusted**	***p***
**Peripheral artery disease**		4174	227			3806	172		
	Remnant cholesterol			1.6 (1.1-2.3)	0.018			1.5 (1.0-2.2)	0.081
	LDL cholesterol			0.9 (0.6-1.2)	0.46			0.9 (0.6-1.3)	0.53
**Myocardial infarction**		3997	227			3806	185		
	Remnant cholesterol			1.7 (1.2-2.5)	0.0054			2.0 (1.3-3.0)	0.00085
	LDL cholesterol			1.0 (0.7-1.4)	0.97			0.9 (0.6-1.3)	0.51
**Ischemic stroke**		3965	219			3806	195		
	Remnant cholesterol			1.5 (1.0-2.2)	0.035			1.5 (1.0-2.2)	0.049
	LDL cholesterol			1.1 (0.8-1.6)	0.64			1.0 (0.7-1.5)	0.94
**Any ASCVD**		3578	478			3806	498		
	Remnant cholesterol			1.6 (1.2-2.0)	0.00051			1.6 (1.2-2.0)	0.00029
	LDL cholesterol			0.9 (0.7-1.1)	0.29			0.9 (0.7-1.1)	0.29

Hazard ratio (95% CI) per double remnant or LDL cholesterol (axis: 1 2 3 4 5)

ESM Fig. 8. Risk of ASCVD per doubling of remnant cholesterol and LDL cholesterol in individuals with diabetes from the Copenhagen General Population Study excluding individuals with diabetes type 1 or ASCVD before baseline.

Results from analyses i) excluding individuals with type 1 diabetes, and ii) excluding individuals with ASCVD before baseline. Cox regression adjusted for age, sex, systolic blood pressure, diastolic blood pressure, smoking status, cumulative smoking, birth year, non-fasting plasma glucose, and LDL cholesterol (in remnant cholesterol analyses) or remnant cholesterol (in LDL cholesterol analyses). ASCVD=Atherosclerotic cardiovascular disease, CI=Confidence interval, LDL=Low-density lipoprotein, No.=Number.

Endpoint	No. of individuals	No. of events		Hazard ratio (95% CI) per double remnant or LDL cholesterol additionally adjusted for LDL triglycerides	*p*
**Peripheral artery disease**	1808	138			
			Remnant cholesterol	0.9 (0.5, 1.6)	0.72
			LDL cholesterol	1.0 (0.6, 1.7)	0.95
**Myocardial infarction**	1714	210			
			Remnant cholesterol	1.6 (1.0, 2.7)	0.048
			LDL cholesterol	0.9 (0.6, 1.4)	0.66
**Ischemic stroke**	1707	195			
			Remnant cholesterol	1.3 (0.8, 2.3)	0.27
			LDL cholesterol	0.9 (0.6, 1.5)	0.72
**Any ASCVD**	1482	378			
			Remnant cholesterol	1.2 (0.9, 1.8)	0.25
			LDL cholesterol	0.8 (0.5, 1.1)	0.16

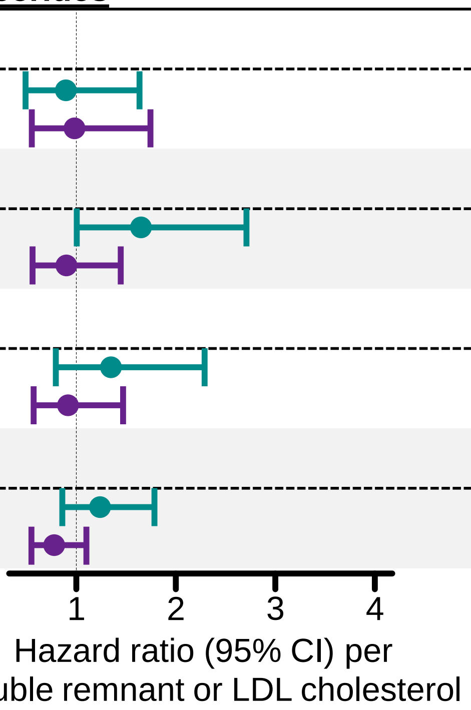

ESM Fig. 9. Risk of ASCVD per doubling of remnant cholesterol and LDL cholesterol in individuals with diabetes from the Copenhagen General Population Study after adjustment for LDL triglycerides.

The Cox model was adjusted for age, sex, systolic blood pressure, diastolic blood pressure, smoking status, cumulative smoking, birth year, non-fasting plasma glucose, LDL triglycerides, and LDL cholesterol (in remnant cholesterol analyses) or remnant cholesterol (in LDL cholesterol analyses). ASCVD=Atherosclerotic cardiovascular disease, CI=Confidence interval, HDL=High-density lipoprotein, LDL=Low-density lipoprotein, No.=Number.

Endpoint		Non-fasting glucose <6.5 mmol/L (<median) No. of individuals	No. of events	Hazard ratio (95% CI) per double remnant or LDL cholesterol, multivariable adjusted	*p*	Non-fasting glucose ≥6.5 mmol/L (≥median) No. of individuals	No. of events	Hazard ratio (95% CI) per double remnant or LDL cholesterol, multivariable adjusted	*p*
Peripheral artery disease		2142	97			2272	139		
	Remnant cholesterol			1.7 (0.9, 3.0)	0.089			1.5 (1.0, 2.4)	0.064
	LDL cholesterol			0.6 (0.4, 1.1)	0.12			1.0 (0.7, 1.6)	0.87
Myocardial infarction		2071	103			2166	131		
	Remnant cholesterol			1.5 (0.8, 2.6)	0.18			2.0 (1.3, 3.3)	0.0036
	LDL cholesterol			0.9 (0.5, 1.6)	0.72			1.1 (0.7, 1.8)	0.59
Ischemic stroke		2037	96			2165	130		
	Remnant cholesterol			1.4 (0.7, 2.5)	0.32			1.6 (1.0, 2.5)	0.057
	LDL cholesterol			1.3 (0.7, 2.5)	0.36			1.0 (0.7, 1.6)	0.92
Any ASCVD		1846	202			1960	296		
	Remnant cholesterol			1.4 (0.9, 2.1)	0.13			1.7 (1.2, 2.3)	0.001
	LDL cholesterol			1.0 (0.6, 1.5)	0.83			0.9 (0.6, 1.2)	0.35

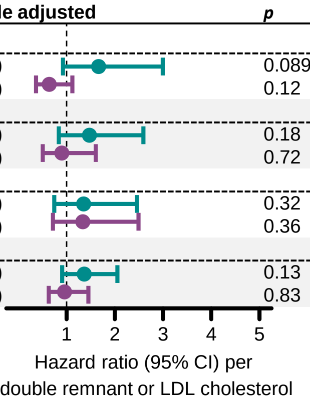

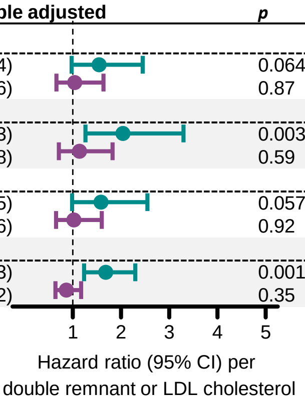

ESM Fig. 10. Risk of ASCVD per doubling of remnant cholesterol and LDL cholesterol in individuals with diabetes from the Copenhagen General Population Study by groups of non-fasting plasma glucose.

Results from i) individuals with non-fasting plasma glucose <6.5 mmol/L (<median), and ii) individuals with non-fasting plasma glucose ≥6.5 mmol/L (≥median). Cox regression adjusted for age, sex, systolic blood pressure, diastolic blood pressure, smoking status, cumulative smoking, birth year, non-fasting plasma glucose, and LDL cholesterol (in remnant cholesterol analyses) or remnant cholesterol (in LDL cholesterol analyses). ASCVD=Atherosclerotic cardiovascular disease, CI=Confidence interval, LDL=Low-density lipoprotein, No.=Number.

Endpoint	No. of individuals	No. of events	No. of events per 1,000 person-years (95% CI): Median level	No. of events per 1,000 person-years (95% CI): Double median level	Hazard ratio (95% CI) per double directly measured remnant or LDL cholesterol, multivariable adjusted	*p*
**Peripheral artery disease**	1808	138				
Remnant cholesterol			9 (8, 11)	10 (7, 13)	1.0 (0.7, 1.5)	0.88
LDL cholesterol			9 (8, 11)	9 (7, 13)	1.3 (0.7, 2.5)	0.37
**Myocardial infarction**	1714	210				
Remnant cholesterol			15 (13, 17)	16 (13, 20)	1.3 (1.0, 1.8)	0.098
LDL cholesterol			15 (13, 17)	12 (9, 16)	0.8 (0.5, 1.2)	0.26
**Ischemic stroke**	1707	195				
Remnant cholesterol			14 (12, 16)	18 (14, 22)	1.6 (1.1, 2.2)	0.0077
LDL cholesterol			14 (12, 16)	14 (10, 19)	0.7 (0.4, 1.2)	0.25
**Any ASCVD**	1482	378				
Remnant cholesterol			33 (30, 37)	38 (32, 44)	1.3 (1.0, 1.7)	0.018
LDL cholesterol			34 (30, 37)	28 (23, 35)	0.8 (0.5, 1.1)	0.12

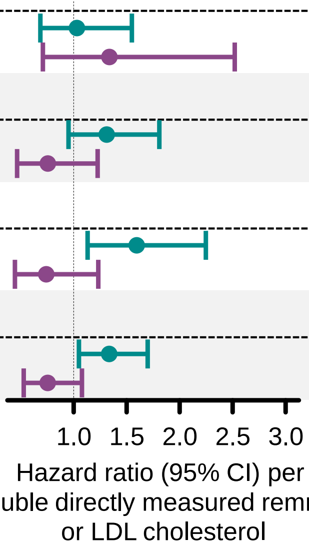

ESM Fig. 11. Risk of ASCVD per doubling of directly measured remnant cholesterol and LDL cholesterol in individuals with diabetes from the Copenhagen General Population Study.

Cox regression adjusted for age, sex, systolic blood pressure, diastolic blood pressure, smoking status, cumulative smoking, birth year, non-fasting glucose, and directly measured LDL cholesterol (in remnant cholesterol analyses) or directly measured remnant cholesterol (in LDL cholesterol analyses). ASCVD=Atherosclerotic cardiovascular disease, CI=Confidence Interval, LDL=Low-density lipoprotein, No.=Number.

Endpoint	No. of individuals	No. of events	No. of events per 1,000 person-years (95% CI): Median level	Double median level	Hazard ratio (95% CI) per double directly measured remnant cholesterol or LDL triglycerides, multivariable adjusted	*p*
**Peripheral artery disease**	1808	138				
	**Remnant cholesterol**		9 (8, 11)	10 (7, 13)	0.8 (0.5, 1.4)	0.48
	**LDL triglycerides**		9 (8, 11)	11 (7, 17)	2.2 (0.7, 6.8)	0.19
**Myocardial infarction**	1714	210				
	**Remnant cholesterol**		15 (13, 17)	16 (13, 20)	1.3 (0.9, 2.0)	0.17
	**LDL triglycerides**		15 (13, 17)	14 (10, 19)	0.9 (0.4, 2.4)	0.87
**Ischemic stroke**	1707	195				
	**Remnant cholesterol**		14 (12, 16)	18 (14, 22)	1.5 (0.9, 2.3)	0.10
	**LDL triglycerides**		14 (12, 16)	19 (14, 27)	1.4 (0.5, 3.6)	0.54
**Any ASCVD**	1482	378				
	**Remnant cholesterol**		33 (30, 37)	38 (32, 44)	1.2 (0.9, 1.6)	0.34
	**LDL triglycerides**		33 (30, 37)	37 (29, 48)	1.6 (0.8, 3.3)	0.17

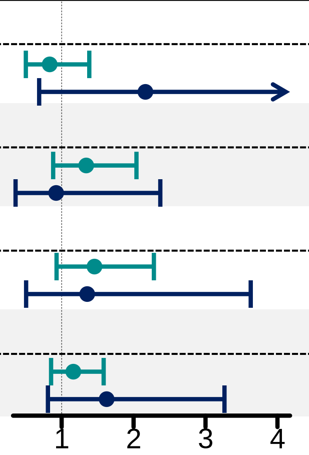

ESM Fig. 12. Risk of ASCVD per doubling of directly measured remnant cholesterol and LDL triglycerides in individuals with diabetes from the Copenhagen General Population Study.

Cox regression adjusted for age, sex, systolic blood pressure, diastolic blood pressure, smoking status, cumulative smoking, birth year, non-fasting plasma glucose, directly measured LDL cholesterol, LDL triglycerides (in remnant cholesterol analyses) or directly measured remnant cholesterol (in LDL triglycerides analyses). ASCVD=Atherosclerotic cardiovascular disease, CI=Confidence Interval, LDL=Low-density lipoprotein, No.=Number.

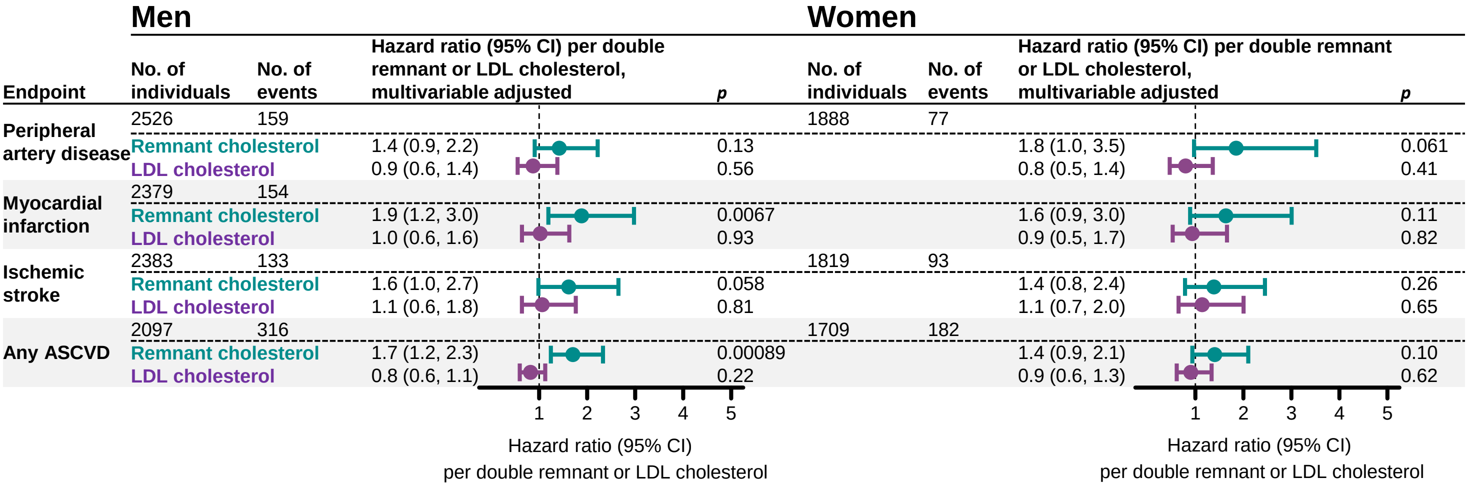

Endpoint		Men: No. of individuals	Men: No. of events	Men: Hazard ratio (95% CI) per double remnant or LDL cholesterol, multivariable adjusted	Men: *p*	Women: No. of individuals	Women: No. of events	Women: Hazard ratio (95% CI) per double remnant or LDL cholesterol, multivariable adjusted	Women: *p*
Peripheral artery disease		2526	159			1888	77		
	Remnant cholesterol			1.4 (0.9, 2.2)	0.13			1.8 (1.0, 3.5)	0.061
	LDL cholesterol			0.9 (0.6, 1.4)	0.56			0.8 (0.5, 1.4)	0.41
Myocardial infarction		2379	154						
	Remnant cholesterol			1.9 (1.2, 3.0)	0.0067			1.6 (0.9, 3.0)	0.11
	LDL cholesterol			1.0 (0.6, 1.6)	0.93			0.9 (0.5, 1.7)	0.82
Ischemic stroke		2383	133			1819	93		
	Remnant cholesterol			1.6 (1.0, 2.7)	0.058			1.4 (0.8, 2.4)	0.26
	LDL cholesterol			1.1 (0.6, 1.8)	0.81			1.1 (0.7, 2.0)	0.65
Any ASCVD		2097	316			1709	182		
	Remnant cholesterol			1.7 (1.2, 2.3)	0.00089			1.4 (0.9, 2.1)	0.10
	LDL cholesterol			0.8 (0.6, 1.1)	0.22			0.9 (0.6, 1.3)	0.62

Hazard ratio (95% CI) per double remnant or LDL cholesterol

ESM Fig. 13. Risk of ASCVD per doubling of remnant cholesterol and LDL cholesterol in individuals with diabetes from the Copenhagen General Population Study in men and women

Results for i) men only, and ii) women only, from Cox regression multivariable adjusted for age, systolic blood pressure, diastolic blood pressure, smoking status, cumulative smoking, non-fasting plasma glucose, birth year, and LDL cholesterol (in remnant cholesterol analyses) or remnant cholesterol (in LDL cholesterol analyses). Number of events per 1,000 person years is from unadjusted Poisson regression. ASCVD=Atherosclerotic cardiovascular disease, CI=Confidence interval, LDL=Low-density lipoprotein, No.=Number.

Group	Endpoint	No. of individuals	No. of events	No. of events per 1,000 person-years: No diabetes	No. of events per 1,000 person-years: Diabetes	Hazard ratio (95% CI) for diabetes versus no diabetes	*p*
**Statin users**	**Peripheral artery disease**	12,418	500	4	8	1.6 (1.3, 1.9)	1×10^{-6}
	**Myocardial infarction**	11,291	527	5	7	1.3 (1.0, 1.5)	0.019
	**Ischemic stroke**	11,126	495	5	6	1.2 (1.0, 1.5)	0.094
	**Any ASCVD**	9322	1008	13	17	1.2 (1.1, 1.4)	0.0043
**Smokers**	**Peripheral artery disease**	18,143	679	4	12	2.3 (1.9, 3.0)	6×10^{-13}
	**Myocardial infarction**	17,965	633	3	7	1.5 (1.1, 2.0)	0.0081
	**Ischemic stroke**	17,799	671	4	9	1.6 (1.2, 2.1)	0.0013
	**Any ASCVD**	17,257	1664	10	23	1.7 (1.4, 2.0)	5×10^{-8}

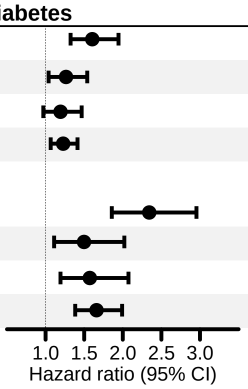

ESM Fig. 14. Excess risk of ASCVD for diabetes in individuals who were using statins and who were smokers, respectively, from the Copenhagen General Population Study.

Cox regression adjusted for age, sex, smoking status, cumulative smoking, and birth year. ASCVD=Atherosclerotic cardiovascular disease, CI=Confidence interval, LDL=Low-density lipoprotein, No.=Number.

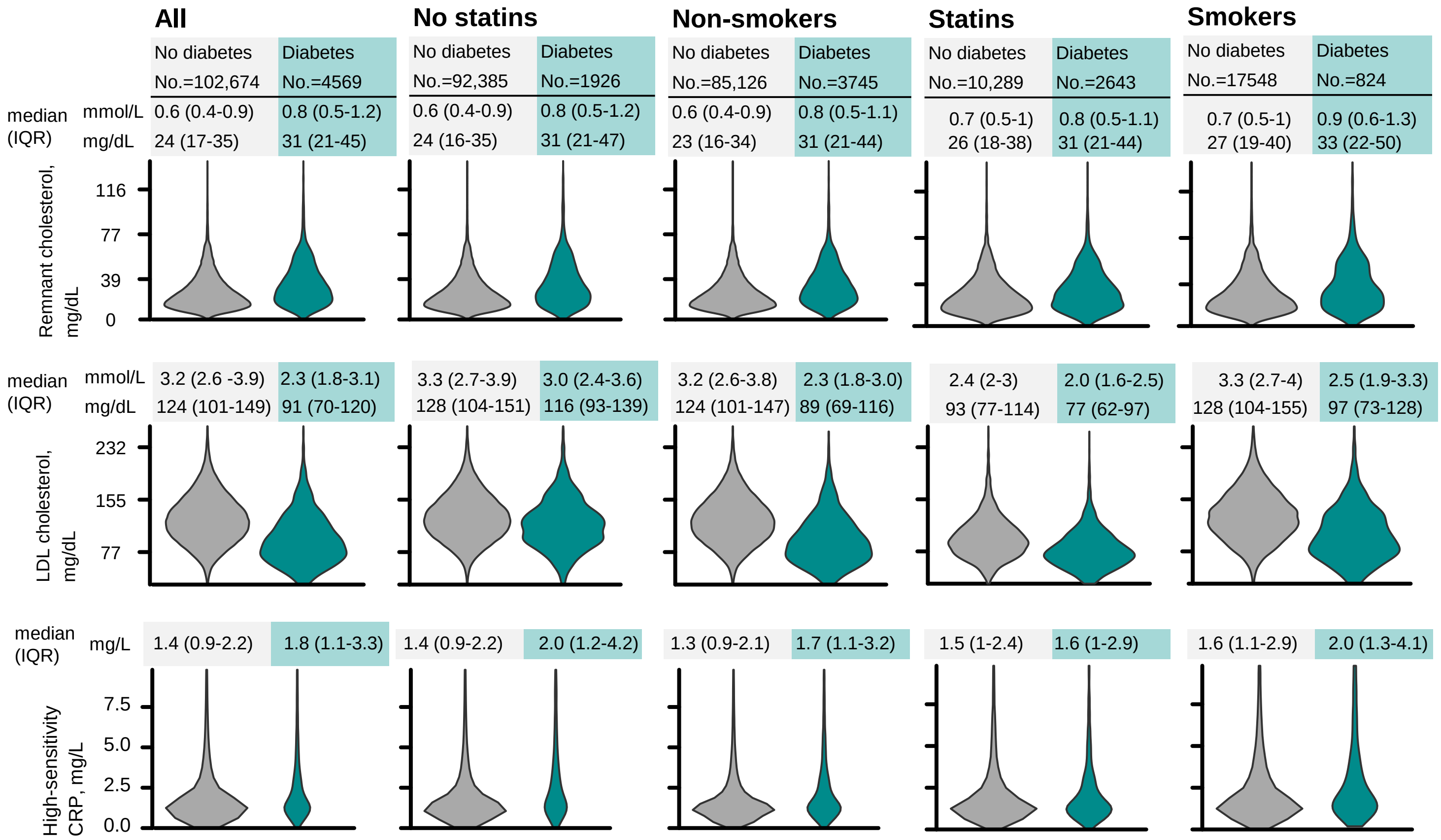

ESM Fig. 15. Violin plots showing densities of remnant cholesterol, LDL cholesterol, and high-sensitivity C-reactive protein in individuals with and without diabetes by statin use and current smoking status, in the Copenhagen General Population Study.

CRP= C-reactive protein, IQR=Interquartile range, LDL=Low-density lipoprotein, No.=Number.

Group	Endpoint	Explanatory factor	Explained excess risk in diabetes, % (95% CI)	*p*
No statins	Peripheral artery disease	Remnant cholesterol	15 (2, 29)	0.029
		LDL cholesterol	0	NA
		Low-grade inflammation	12 (0, 26)	0.099
	Myocardial infarction	Remnant cholesterol	28 (9, 48)	0.0047
		LDL cholesterol	0	NA
		Low-grade inflammation	22 (2, 41)	0.030
	Ischemic stroke	Remnant cholesterol	15 (0, 36)	0.15
		LDL cholesterol	0	NA
		Low-grade inflammation	39 (10, 67)	0.0081
	Any ASCVD	Remnant cholesterol	19 (7, 30)	0.0014
		LDL cholesterol	0	NA
		Low-grade inflammation	21 (9, 34)	0.00064
Non-smokers	Peripheral artery disease	Remnant cholesterol	19 (10, 28)	5×10^{-5}
		LDL cholesterol	0	NA
		Low-grade inflammation	8 (2, 13)	0.0047
	Myocardial infarction	Remnant cholesterol	20 (11, 29)	2×10^{-5}
		LDL cholesterol	0	NA
		Low-grade inflammation	7 (1, 13)	0.017
	Ischemic stroke	Remnant cholesterol	8 (0, 21)	0.28
		LDL cholesterol	0	NA
		Low-grade inflammation	26 (13, 40)	9×10^{-5}
	Any ASCVD	Remnant cholesterol	15 (9, 22)	5×10^{-6}
		LDL cholesterol	0	NA
		Low-grade inflammation	12 (7, 16)	1×10^{-6}

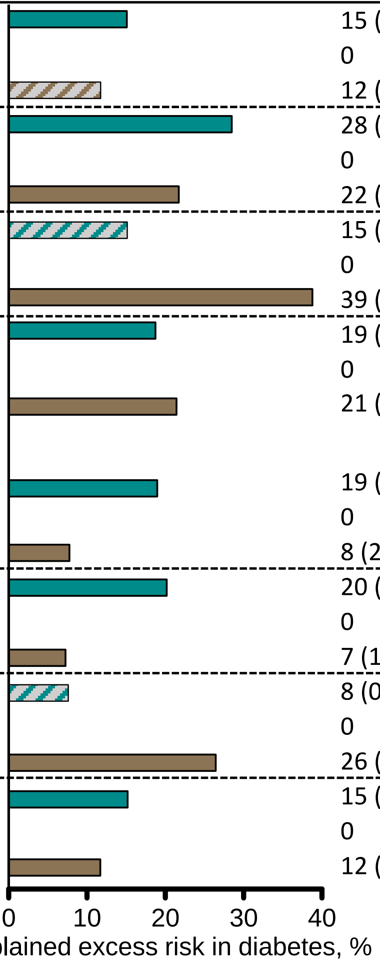

ESM Fig. 16. Explained fraction of excess risk of ASCVD in diabetes by remnant cholesterol LDL cholesterol, and low-grade inflammation measured as elevated high-sensitivity C-reactive protein in individuals not using statins and in non-smokers from the Copenhagen General Population Study.

LDL cholesterol was lower in individuals with diabetes than in individuals without diabetes, why estimates for elevated LDL cholesterol were not applicable. Adjusted for age, sex, smoking status (except for analyses in non-smokers), cumulative smoking, and birth year. Estimates below 0 were truncated to 0%. Striped bars indicate P>0.05. ASCVD=Atherosclerotic cardiovascular disease, CI=Confidence interval, LDL=Low-density lipoprotein. NA=Not applicable

Glucose status	Endpoint	No. of individuals	No. of events	No. of events per 1,000 person-years: No diabetes	No. of events per 1,000 person-years: Diabetes	Hazard ratio (95% CI) for diabetes versus no diabetes	*p*
**Diabetes with non-fasting glucose <6.5 mmol/L (<median)**	**Peripheral artery disease**	104,138	1431	1	5	2.0 (1.6-2.5)	3×10^{-11}
	**Myocardial infarction**	102,842	2410	2	6	1.5 (1.2-1.8)	0.00020
	**Ischemic stroke**	101,617	2623	3	6	1.2 (1.0-1.4)	0.13
	**Any ASCVD**	99,199	5545	6	14	1.3 (1.1-1.5)	0.00084
**Diabetes with non-fasting glucose ≥6.5 mmol/L (≥median)**	**Peripheral artery disease**	104,264	1473	1	7	3.0 (2.5-3.6)	9×10^{-35}
	**Myocardial infarction**	102,933	2438	2	7	1.8 (1.5-2.2)	6×10^{-11}
	**Ischemic stroke**	101,741	2657	3	7	1.6 (1.4-2.0)	5×10^{-8}
	**Any ASCVD**	99,309	5639	6	19	1.9 (1.7-2.2)	3×10^{-28}

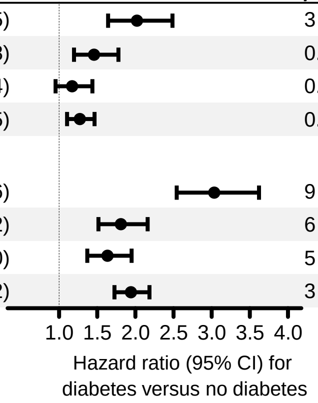

ESM Fig. 17. Excess risk of ASCVD conferred by diabetes with non-fasting plasma glucose <6.5 mmol/L and diabetes with non-fasting plasma glucose ≥6.5 mmol/L, respectively, from the Copenhagen General Population Study.

For both diabetes with non-fasting plasma glucose <6.5 mmol/L and diabetes with non-fasting plasma glucose ≥6.5 mmol/L, excess risk is relative to all individuals without diabetes. Cox regression adjusted for age, sex, smoking status, cumulative smoking, and birth year. ASCVD=Atherosclerotic cardiovascular disease, CI=Confidence interval, LDL=Low-density lipoprotein, No.=Number.

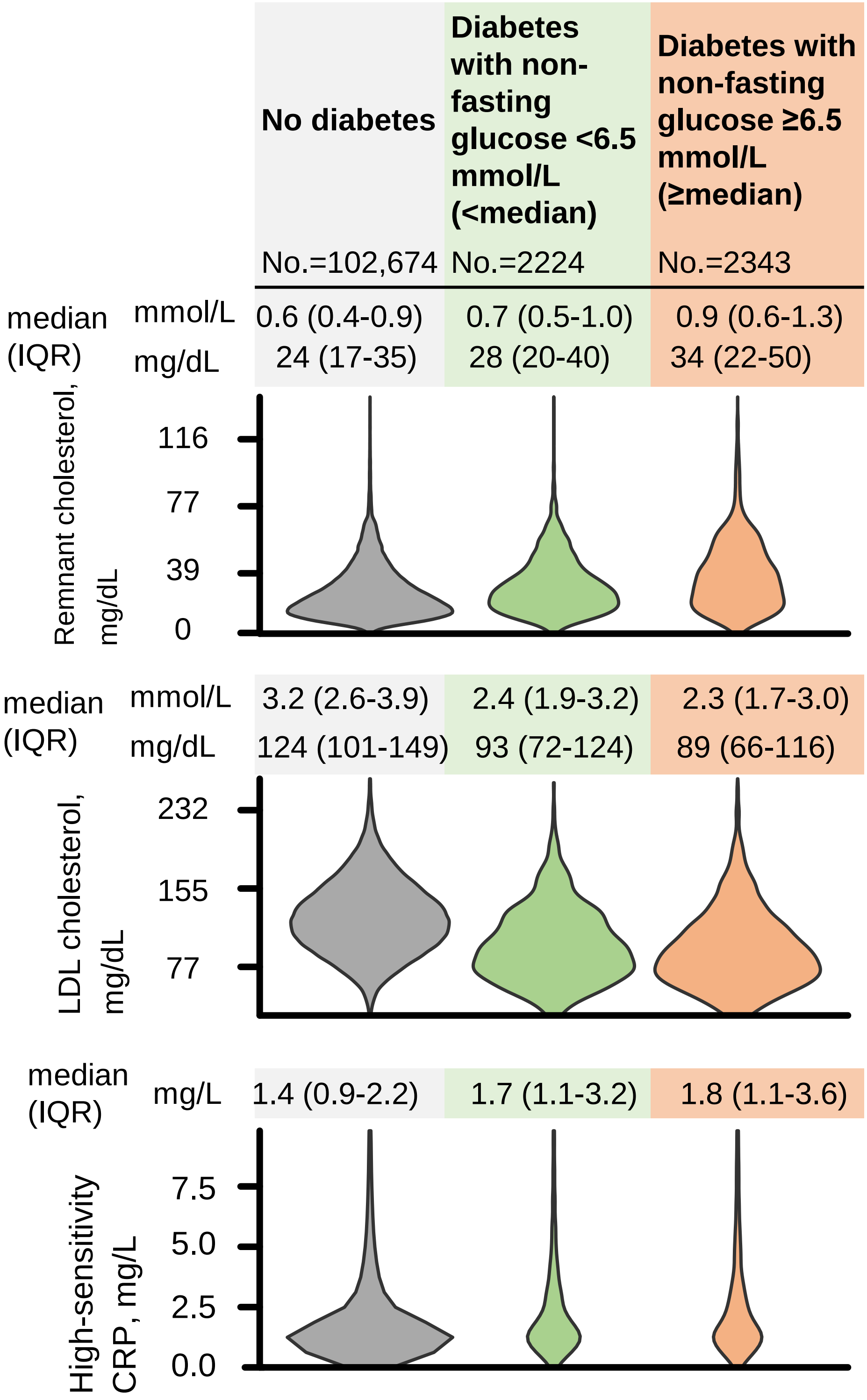

ESM Fig. 18. Violin plots showing densities of remnant cholesterol, LDL cholesterol, and high-sensitivity C-reactive protein in individuals without diabetes, individuals with diabetes and non-fasting plasma glucose <6.5 mmol/L, and individuals with diabetes and non-fasting plasma glucose ≥6.5 mmol/L, respectively, in the Copenhagen General Population Study.

CRP= C-reactive protein, IQR=Interquartile range, LDL=Low-density lipoprotein, No.=Number.

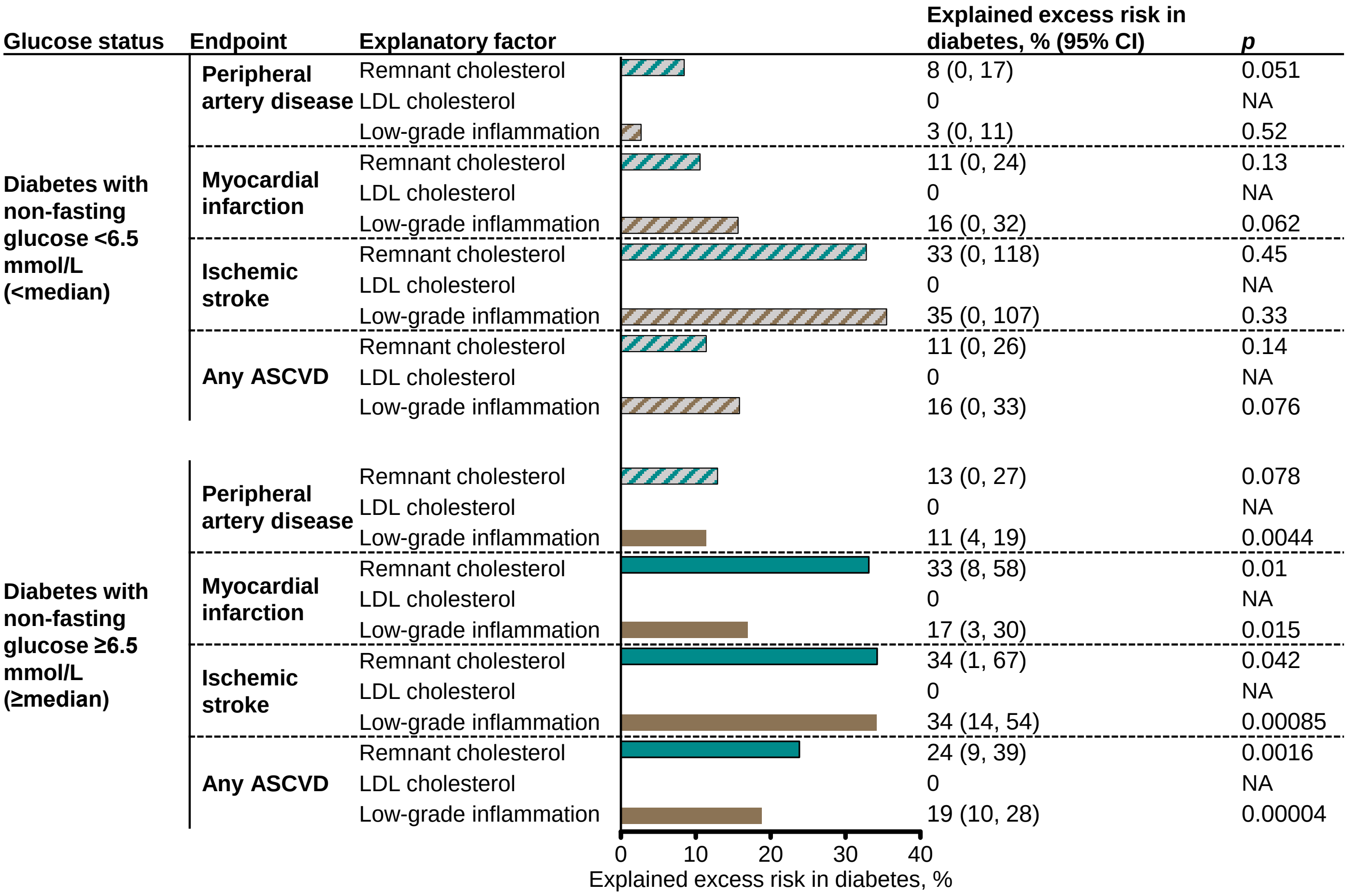

ESM Fig. 19. Fraction of excess risk of ASCVD <u>conferred by diabetes with non-fasting plasma glucose <6.5 mmol/L and by diabetes with non-fasting plasma glucose ≥6.5 mmol/L</u> explained by remnant cholesterol, LDL cholesterol, and low-grade inflammation measured according to elevated high-sensitivity C-reactive protein from the Copenhagen General Population Study.

LDL cholesterol was lower in individuals with diabetes than in individuals without diabetes, why estimates for elevated LDL cholesterol were not applicable. Adjusted for age, sex, smoking status (except for analyses in non-smokers), cumulative smoking, and birth year. Estimates below 0 were truncated to 0%. Striped bars indicate P>0.05. ASCVD=Atherosclerotic cardiovascular disease, CI=Confidence interval, LDL=Low-density lipoprotein. NA=Not applicable
